# UGDH Lactylation Aggravates Osteoarthritis by Suppressing Glycosaminoglycan Synthesis and Orchestrating Nucleocytoplasmic Transport to Activate MAPK Signaling

**DOI:** 10.1002/advs.202413709

**Published:** 2025-03-27

**Authors:** Weiren Lan, Xueman Chen, Huai Yu, Jianzhao Ruan, Jingliang Kang, Xiaoyu Nie, Yumei Cao, Su'an Tang, Changhai Ding

**Affiliations:** ^1^ Clinical Research Centre Zhujiang Hospital Southern Medical University Guangzhou 510280 China; ^2^ Breast Tumor Center Sun Yat‐sen Memorial Hospital Sun Yat‐sen University Guangzhou 510120 China; ^3^ Department of Spinal Surgery Orthopedic Medical Center Zhujiang Hospital Southern Medical University Guangzhou 510280 China; ^4^ Institute of Exercise and Rehabilitation Science Zhujiang Hospital Southern Medical University Guangzhou 510280 China; ^5^ Menzies Institute for Medical Research University of Tasmania Hobart Tasmania Australia

**Keywords:** chondrocytes, lactate, lactylation, osteoarthritis, udp‐glucose dehydrogenase

## Abstract

Osteoarthritis (OA) progression is closely related to dysregulated glycolysis. As the primary metabolite of glycolysis, lactate plays a detrimental role in OA. However, how lactate exacerbates OA process remains unclear. Here, this study revealed that lactate levels are elevated in the synovial fluid of OA patients and IL‐1β‐treated human primary chondrocytes, promoting protein pan‐lactylation. Functionally, hyper‐lactylation exacerbates chondrocytes extracellular matrix (ECM) degradation and cell apoptosis in vitro and in vivo. Moreover, UDP‐glucose dehydrogenase (UGDH) is proven to be the key lactylated protein in lactate‐treated chondrocytes, which undergoes lactylation at lysine 6 (K6). Lactylated UGDH repressed its enzymatic activity, reducing glycosaminoglycan synthesis and disregulating its nuclear‐cytoplasmic distribution. Mechanistically, K6 lactylation of UGDH impedes the interaction of UGDH and signal transducer and activator of transcription 1 (STAT1), thus promoting the transcription of mitogen‐activated protein kinase kinase kinase 8 (MAP3K8) and activating the MAPK signaling pathway. Importantly, in vitro and in vivo treatment with A485, a specific acyltransferase P300 inhibitor, suppressed UGDH lactylation and rescued chondrocytes ECM degradation and OA progression. These findings uncover a new mechanism underlying OA pathogenesis and highlight the potential of targeting UGDH lactylation as a novel therapeutic strategy for OA.

## Graphical abstract

Lactylation of UDP‐glucose dehydrogenase (UGDH) represses its enzymatic activity and causes glycosaminoglycans loss. In addition, UGDH lactylation facilitates its interaction with chromosome region maintenance 1 (CRM1) and promotes the nucleocytoplasmic transport. In nucleus, the interaction of lactylated UGDH and signal transducer and activator of transcription 1 (STAT1) is disrupted, which promotes the binding of STAT1 and mitogen‐activated protein kinase kinase kinase 8 (MAP3K8) promoter, ultimately results in the transcription of MAP3K8 and activation of MAPK pathway.

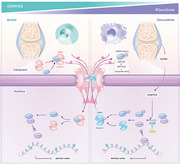

## 1 Introduction

Osteoarthritis (OA) is the most common musculoskeletal disease in humans, impacting at least 654 million individuals globally with increasing prevalence.^[^ ^1^ ^]^ Currently, it poses a huge health and economic burden, especially on the elderly.^[^ ^2^ ^]^ The pathogenesis of OA encompasses the activation of diverse inflammatory signaling cascades, notably including the NF‐κB and mitogen‐activated protein kinase (MAPK) pathways,^[^ ^3^, ^4^ ^]^ which regulate the release of pro‐inflammatory cytokines and matrix metalloproteinases (MMPs), inducing cell apoptosis, thereby driving OA progression.^[^ ^5^ ^]^ Additionally, OA development is influenced by metabolic, genetic, and mechanical factors.^[^ ^2^ ^]^ The complexity and heterogeneity of OA pathogenesis contribute to the current lack of effective drugs to halt OA progression, highlighting the importance of a thorough understanding of its underlying mechanisms.

Articular cartilage degradation is a hallmark of OA. This avascular tissue provides a naturally hypoxic microenvironment for chondrocytes, where oxidative phosphorylation is relatively limited. Glycolysis emerges as an efficient and swift pathway for ATP production, thereby serving as the primary energy‐generating mechanism for chondrocytes.^[^ ^6^, ^7^ ^]^ In the inflammatory environment of OA, chondrocytes undergo metabolic reprogramming, enhancing glycolysis to meet heightened energy demands. Lactate, a primary glycolytic metabolite, serves as both a major energy source and a signaling molecule, ultimately, influencing disease progression.^[^ ^8^, ^9^ ^]^ It has been found that the level of lactate in the synovial fluid of OA patients is significantly elevated,^[^ ^10^ ^]^ correlating with increased reactive oxygen species (ROS) synthesis, enhanced MMPs expression, and suppressed anabolic genes such as collagen II, leading to extracellular matrix (ECM) degradaiton.^[^ ^11^ ^]^ Inhibiting glycolysis and reducing lactate concentration have shown promise in delaying OA progression.^[^ ^10^ ^]^ Thus, further exploration of the underlying molecular mechanisms in OA is critical.

Recent studies have suggested that lactate may exert its biological activity through protein lactylation.^[^ ^12^ ^]^ Lactylation plays an important epigenetic regulatory role in various biological processes.^[^ ^12^ ^]^ As a novel post‐translational modification (PTM) affecting both histones and non‐histones, lactylation precisely regulates gene expression through histones. For instance, lactylation of histone H4K12 promotes the transcription of pyruvate kinase M2 (PKM2) and exacerbates microglial dysfunction in Alzheimer's disease.^[^ ^13^ ^]^ In addition, lactylation influences the interaction between proteins, such as the lactylation of α‐myosin heavy chain (α‐MHC) at lysine 1897 (K1897), which maintains its interaction with Titin, thereby protecting the damaged myocardium.^[^ ^14^ ^]^ Despite the crucial role of lactate in OA progression, the contribution of lactate‐driven lactylation to OA and the underlying mechanisms remain unclarified.

Here, we detected the roles of lactylation in OA. We further identified that UDP‐glucose dehydrogenase (UGDH) was the key factor of lactylation‐driven OA by regulating glycosaminoglycan (GAGs) synthesis, ECM metabolism and chondrocyte apoptosis. Lactylation of UGDH repressed its enzymatic activity and regulated its nuclear‐cytoplasmic distribution, led to activation of MAPK pathway and ultimately accelerated OA progression. Targeting lactylation of UGDH represents a novel therapeutic approach for OA treatment.

## 2 Results

### 2.1 Pan‐Lactylation is Increased in OA Articular Cartilage and IL‐1β‐Treated Chondrocytes

**Figure 1:**
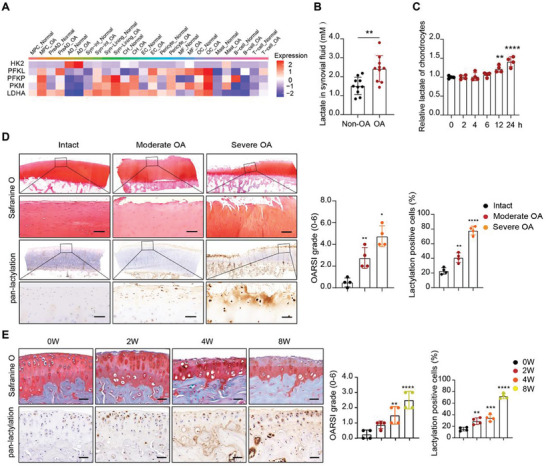
Pan‐lactylation is increased in OA articular cartilage and IL‐1β‐treated chondrocytes. A) Glycolysis related genes expression in joints of non‐OA donors and OA patients according to single‐cell RNA sequencing data. B) Relative lactate production of synovial fluid in non‐OA (*n* = 10 per group) and OA (*n* = 10 per group) patients. C) Relative lactate production of chondrocytes with IL‐1β (10 ng ml^−1^) treatment for 0, 2, 4, 6, 12, and 24 h. D) Safranin O staining (top panel) and pan‐lactylation immunohistochemistry (IHC) staining (bottom panel) of the cartilage from OA patients (4 samples per group). Scale bars, 50 µm. E) Safranin O and Fast Green staining (SOFG) staining (top panel) and pan‐lactylation IHC staining (bottom panel) of the cartilage from DMM‐induced OA mice at 0, 2, 4, and 8 weeks (4 samples per group). Scale bars, 50 µm. Data were presented as mean ± SD and analyzed by Student's t‐test (B) or One‐way analysis of variance (ANOVA) with Dunnett's post hoc test (C,D,E). N represents the number of independent repeated experiments or sample size. Osteoarthritis Research Society International (OARSI) grade were shown as mean ± 95% confidence interval (CI) and Mann‐Whitney U test for statistical analysis (D,E). NS: no significance, *p < 0.05, **p < 0.01, ***p < 0.001, and ****p < 0.0001.

To investigate the metabolic reprogramming in OA, we re‐analyzed glycolysis‐related genes expression based on human knee joint single‐cell transcriptomic data.^[^ ^15^ ^]^ Here, the rate‐limiting glycolytic enzymes, including hexokinases 2 (HK2), phosphofructokinases (PFK), pyruvate kinase M (PKM), and the key enzyme for lactate synthesis, lactate dehydrogenase A (LDHA), were significantly elevated in various cells of joint tissue in OA, especially in precursor adipocytes (PreAD), adipocytes (AD), synovial intermediate fibroblast (Syn‐int), synovial lining layer fibroblast (Syn‐Lining) and osteoclasts (OC) (**Figure** **1**A). We further detected the concentration of lactate in synovial fluid and found that lactate was markedly increased in synovial fluid from OA patients (Figure 1B). Interestingly, lactate was also increased in the culture medium of inflamed chondrocytes treated by IL‐1β (Figure 1C). These data show that lactate is upregulated in OA joints, suggesting the potential role of lactate in OA progression.

To explore the expression pattern of pan‐lactylation in cartilage induced by lactate, we further conducted immunohistochemistry (IHC) assay with pan‐lactylation antibody to investigate the percentage of lactylation positive cells in human and mouse OA cartilage tissues. Pan‐lactylation antibody can specifically recognize all lactylated proteins in cells. Compared with the intact cartilage, the percentage of lactylation positive cells increased with the degree of cartilage destruction from OA patients (Figure 1D). Next, we established experimental OA mouse model by destabilization of the medial meniscus (DMM) surgery (Figure 1E). Consistently, protein pan‐lactylation within cartilage was also elevated in post‐traumatic OA mice joint (Figure 1E). Moreover, we found that the level of pan‐lactylation in chondrocyte was gradually increased with time upon IL‐1β stimulation (Figure S1A, Supporting Information). As showed in immunofluorescence assay, lactylated proteins were increased and located in both cytoplasm and nucleus (Figure S1B, Supporting Information). Collectively, our data demonstrate that pan‐lactylation is increased in OA articular cartilage, indicating that the detrimental role of hyper‐lactylation in OA pathogenesis.

### 2.2 Hyper‐Lactylation Aggravates Chondrocyte Extracellular Matrix (ECM) Degradation and Cell Apoptosis

To investigate the role of lactylation in OA, lactate was utilized to elevate the level of protein pan‐lactylation in human primary chondrocytes. This increase in pan‐lactylation induced by lactate led to a significant upregulation of catabolic molecules, such as MMP3, MMP13 and a disintegrin and metalloproteinase with thrombospondin motifs 4 (ADAMTS4), and a marked reduction of anabolic molecules Collagen II and Aggrecan (Figure S2A,B, Supporting Information). In addition, lactate‐treated chondrocytes expressed more apoptosis‐related proteins, including cleaved‐caspase 3 and Bax, but less anti‐apoptotic protein Bcl‐2 (Figure S2C, Supporting Information). Immunofluorescence assay further indicated a notable increase in the presence of early apoptotic cells labeled by Annexin V (green) and late apoptotic or necrotic cells labeled by PI (red) following lactate treatment (Figure S2D, Supporting Information).

Sodium dichloroacetate (DCA) has been reported to reduce lactate production by inhibiting the activity of pyruvate dehydrogenase kinase, subsequently decreases lactylation level.^[^ ^16^ ^]^ To further assess the impact of lactylation on ECM degradation and apoptosis, DCA was used to inhibit lactate synthesis and protein pan‐lactylation. Results showed that DCA decreased the lactate synthesis of chondrocytes (Figure S2E, Supporting Information). In the presence of IL‐1β, DCA‐mediated inhibition of pan‐lactylation significantly reduced the expression levels of MMP3, MMP13, and ADAMTS4, while enhancing the expression of Collagen II and Aggrecan, remarkably (Figure S2F,G, Supporting Information). Regarding apoptosis, DCA treatment significantly inhibited the expression of cleaved‐caspase 3 and Bax, contrary to the increase expression of Bcl‐2 (Figure S2H, Supporting Information). Consistently, DCA reduced the rate of apoptotic cells, as showed by immunofluorescence assay (Figure S2I, Supporting Information). Taken together, our data demonstrate that hyper‐lactylation promotes ECM degradation and chondrocyte apoptosis in OA.

### 2.3 Hyper‐Lactylation Aggravates OA Progression In Vivo

**Figure 2:**
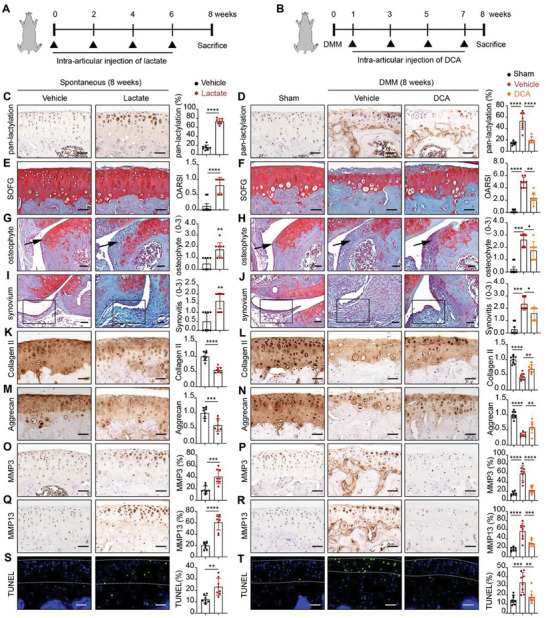
Hyper‐lactylation aggravates OA progression in vivo. A,B) Schematic diagram of animals experiment design. C) Immunohistochemistry (IHC) of pan‐lactylation of articular cartilage in non‐surgical mice with lactate or PBS (*n* = 8 per group). Scale bars, 50 µm. D) IHC of pan‐lactylation of articular cartilage in sham, DMM induced OA mice with DCA or PBS (*n* = 8 per group). Scale bars, 50 µm. E) Safranin O and Fast Green staining (SOFG) of articular cartilage in non‐surgical mice with lactate or PBS. OARSI grade was measured according to SOFG staining. Scale bars, 50 µm. F) SOFG of articular cartilage in sham, DMM induced OA mice with DCA or PBS. OARSI grade was measured according to SOFG staining. Scale bars, 50 µm. G) SOFG of osteophyte (arrow mark) in non‐surgical mice with lactate or PBS. Osteophyte score for analysis of osteophyte formation. Scale bars, 100 µm. H) SOFG of osteophyte (arrow mark) in sham, DMM induced OA mice with DCA or PBS. Osteophyte score for analysis of osteophyte formation. Scale bars, 100 µm. I) SOFG of synovium (rectangle mark) in non‐surgical mice with lactate or PBS. Synovium score for analysis of synovitis. Scale bars, 100 µm. J) SOFG of synovium (rectangle mark) in sham, DMM induced OA mice with DCA or PBS. Synovium score for analysis of synovitis. Scale bars, 100 µm. IHC of Collagen II K), Aggrecan M), MMP3 O), and MMP13 Q) of articular cartilage in non‐surgical mice with lactate or PBS. Scale bars, 50 µm. IHC of Collagen II L), Aggrecan N), MMP3 P) and MMP13 R) of articular cartilage in sham, DMM induced OA mice with DCA or PBS. Scale bars, 50 µm. S) TUNEL analysis of articular cartilage in non‐surgical mice with lactate or PBS. Green fluorescence represented TUNEL positive cells. Blue fluorescence (DAPI) represented all cells. Scale bars, 100 µm. T) TUNEL analysis of articular cartilage in sham, DMM induced OA mice with DCA or PBS. Green fluorescence represented TUNEL positive cells. Blue fluorescence (DAPI) represented all cells. Scale bars, 100 µm. Data were presented as mean ± SD and analyzed by Student's t‐test (**C,K,M,O,Q,S**) or One‐way analysis of variance (ANOVA) with Dunnett's post hoc test (**D,L,N,P,R,T**). N represents the number of sample size. Scores of OARSI, osteophyte and synovitis were shown as mean ± 95% CI and Mann‐Whitney U test for statistical analysis (**E**,**F,G,H,I,J**).NS: no significance, *p < 0.05, **p < 0.01, ***p < 0.001, and ****p < 0.0001.

To further examine the biological role of lactylation in OA development and progression, we performed articular injection in non‐surgical mice using lactate (with PBS as the control) and DMM surgery mice using DCA (with PBS as the control) over an 8‐week period (**Figure** **2**A,B). IHC analyses revealed that elevated pan‐lactylation level due to lactate (Figure 2C). In contrast, DCA treatment significantly reduced cartilage pan‐lactylation levels (Figure 2D). Results from Safranine O and Fast Green staining (SOFG) showed that lactate injections spontaneously led to severe cartilage destruction, osteophyte formation and synovitis in non‐surgical mice (Figure 2E,G,I). Conversely, DCA treatment substantially alleviated cartilage loss, osteophyte formation and synovitis in experimental OA mice (Figure 2F,H,J). In addition, IHC analyses revealed that lactate injections led to downregulation of Collagen II and Aggrecan in cartilage (Figure 2K,M), alongside the upregulation of MMP3 and MMP13 (Figure 2O,Q). In contrast, DCA treatment significantly increased Collagen II and Aggrecan expression (Figure 2L,N), and decreased MMP3 and MMP13 expression in OA mice (Figure 2P,R). In vivo apoptosis detection showed that green fluorescence marked apoptotic cells were increased in lactate‐treated mice (Figure 2S), whereas DCA treatment reduced the rate of apoptotic cells in cartilage (Figure 2T). These findings suggest that hyper‐lactylation exacerbates OA progression by promoting ECM degradation and chondrocyte apoptosis.

### 2.4 UGDH Lactylation Accounts for ECM Degradation and Chondrocyte Apoptosis

**Figure 3:**
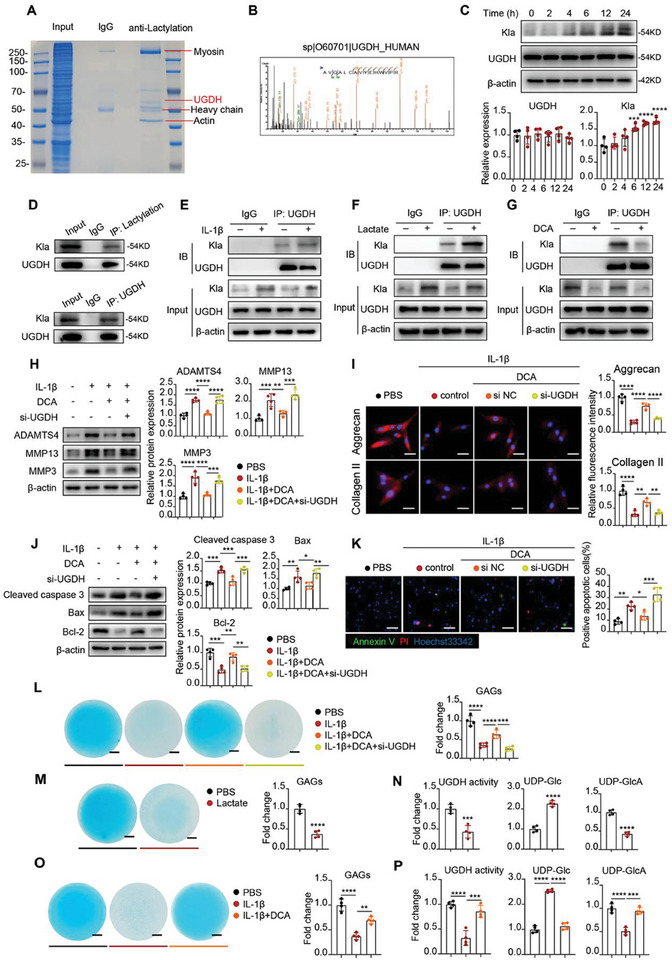
UGDH lactylation accounts for ECM degradation and chondrocyte apoptosis. A) Coomassie bright blue stain of lactylated proteins lysate after CoIP test with lactylation antibody and SDS‐PAGE (10% gel). B) The specific peptide of UGDH in lactylated bands was identified by mass spectrometry analysis. C) Western blot analysis of UGDH and lactylation (Kla) of UGDH band site in chondrocytes with IL‐1β (10 ng ml^−1^) treatment for 0, 2, 4, 6, 12, and 24 h. The data were normalized to β‐actin. D) COIP of UGDH by lactylation antibody and reverse verification of lactylation in UGDH band site by UGDH antibody. E‐G) Western blot of UGDH lactylation in chondrocytes with IL‐1β (10 ng ml^−1^) (E) or lactate (25 mm) (F) or DCA (20 mm) (G) treatment for 24 h. H) Western blot of MMP3, MMP13 and ADAMTS4 in chondrocytes with PBS (control group) or IL‐1β (10 ng ml^−1^) or IL‐1β (10 ng ml^−1^) + DCA (20 mm) or IL‐1β (10 ng ml^−1^) + DCA (20 mm) + si‐UGDH (UGDH siRNA) treatment. The data were normalized to β‐actin. I) Immunofluorescence staining of Collagen II and Aggrecan in chondrocytes with PBS (control group) or IL‐1β (10 ng ml^−1^) or IL‐1β (10 ng ml^−1^) + DCA (20 mm) or IL‐1β (10 ng ml^−1^) + DCA (20 mm) + si‐UGDH (UGDH siRNA) treatment. Scale bar, 25 µm. J) Western blot of cleaved‐caspase 3, Bax and Bcl‐2 in chondrocytes with PBS (control group) or IL‐1β (10 ng ml^−1^) or IL‐1β (10 ng ml^−1^) + DCA (20 mm) or IL‐1β (10 ng ml^−1^) + DCA (20 mm) + si‐UGDH (UGDH siRNA) treatment. The data were normalized to β‐actin. K) Representative immunofluorescence images of Annexin V (green) and PI (red) in chondrocytes with PBS (control group) or IL‐1β (10 ng ml^−1^) or IL‐1β (10 ng ml^−1^) + DCA (20 mm) or IL‐1β (10 ng ml^−1^) + DCA (20 mm) + si‐UGDH (UGDH siRNA) treatment. Scale bar, 100 µm. L) Alcian blue staining and ELISA assay of GAGs in chondrocytes with PBS (control group) or IL‐1β (10 ng ml^−1^) or IL‐1β (10 ng ml^−1^) + DCA (20 mm) or IL‐1β (10 ng ml^−1^) + DCA (20 mm) + si‐UGDH (UGDH siRNA) treatment. M) Alcian blue staining and ELISA assay of GAGs in chondrocytes with lactate treatment. N) UGDH activity, UDP‐Glc and UDP‐GlcA concentrations measurements in chondrocytes after lactate treatment. O) Alcian blue staining and ELISA assay of GAGs in chondrocytes with DCA (20 mm) treatment in the presence of IL‐1β. P) UGDH activity, UDP‐Glc and UDP‐GlcA concentrations measurements in chondrocytes with DCA (20 mm) treatment in the presence of IL‐1β. All data were presented as mean ± SD and analyzed by Student's t‐test or One‐way analysis of variance (ANOVA) with Dunnett's post hoc test. N represents the number of independent repeated experiments. NS: no significance, *P < 0.05, **P < 0.01, ***P < 0.001, and ****P < 0.0001.

We next explored the specific lactylated functional protein in lactate‐mediated OA. Utilizing a pan‐lactylation antibody, we performed immunoprecipitation within human chondrocytes proteins, followed by liquid chromatography and mass spectrometry (LC‐MS) analysis of enrich lactylated peptides. As a result, we found a significant enrichment of lactylated peptides from UDP‐glucose dehydrogenase (UGDH) (**Figure** **3**A,B), which displayed clear lactylation modification. This suggests that UGDH might be the key lactylated protein in chondrocytes. Notably, the lactylation level (Kla) of UGDH was significantly increased upon IL‐1β stimulation, while total UGDH expression remained constant (Figure 3C). We further confirmed that UGDH could be lactylated in human chondrocytes (Figure 3D). Consistently, UGDH lactylation level was enhanced by IL‐1β or lactate stimulation, and decreased by DCA treatment (Figure 3E–G). Intriguingly, siRNA‐mediated UGDH knockdown significantly abrogated the protective effects of DCA in IL‐1β‐treated chondrocytes, as shown in promoting ECM degradation and cell apoptosis (Figure 3H–K). These findings indicate that UGDH is required for maintaining chondrocytes homeostasis and the change of UGDH lactylation levels could be critical for chondrocytes degradation.

**Figure 4:**
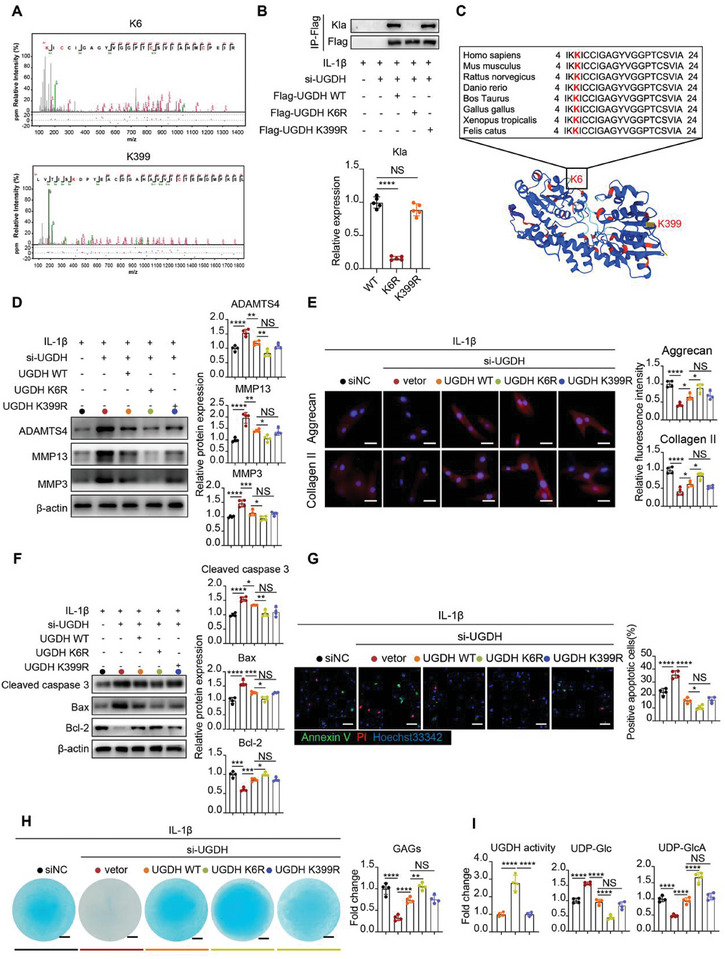
K6 and K399 residues are the lactylated lysine sites in UGDH. A) Lactylated sites of UGDH were identified by liquid chromatography and mass spectrometry analysis. B) Lactylation (Kla) assay of WT UGDH or K6 and K399 sites mutant in UGDH. Chondrocytes were transfected with Flag‐UGDH WT or Flag‐UGDH K6R or Flag‐UGDH K399R plasmids. Flag tagged UGDH was purified by Anti‐Flag antibody. C) K6 site (marked as red) of UGDH is highly conserved. The sequences (4^th^‐24^th^ amino acids) around UGDH K6 site from different species were aligned. D) Western blot analysis of MMP3, MMP13 and ADAMTS4 in chondrocytes with Flag‐UGDH WT or Flag‐UGDH K6R or Flag‐UGDH K399R plasmids transfection in the presence of IL‐1β (10 ng ml^−1^) and UGDH siRNA. The data were normalized to β‐actin. E) Immunofluorescence staining of Collagen II and Aggrecan in chondrocytes with Flag‐UGDH WT or Flag‐UGDH K6R or Flag‐UGDH K399R plasmids transfection in the presence of IL‐1β (10 ng ml^−1^) and UGDH siRNA. Scale bar, 25 µm. F Western blot of cleaved‐caspase 3, Bax and Bcl‐2 in chondrocytes with Flag‐UGDH WT or Flag‐UGDH K6R or Flag‐UGDH K399R plasmids transfection in the presence of IL‐1β (10 ng ml^−1^) and UGDH siRNA. The data were normalized to β‐actin. G) Representative immunofluorescence images of Annexin V (green) and PI (red) in chondrocytes with Flag‐UGDH WT or Flag‐UGDH K6R or Flag‐UGDH K399R plasmids transfection in the presence of IL‐1β (10 ng ml^−1^) and UGDH siRNA. Scale bar, 100 µm. H) Alcian blue staining and ELISA assay of GAGs in chondrocytes with Flag‐UGDH WT or Flag‐UGDH K6R or Flag‐UGDH K399R plasmids transfection in the presence of IL‐1β (10 ng ml^−1^) and UGDH siRNA. I) UGDH activity, UDP‐Glc and UDP‐GlcA concentrations measurements in chondrocytes with Flag‐UGDH WT or Flag‐UGDH K6R or Flag‐UGDH K399R plasmid transfection in the presence of IL‐1β (10 ng ml^−1^) and UGDH siRNA. All data were presented as mean ± SD and analyzed by One‐way analysis of variance (ANOVA) with Dunnett's post hoc test. N represents the number of independent repeated experiments. NS: no significance, *P < 0.05, **P < 0.01, ***P < 0.001, and ****P < 0.0001.

Compelling studies reported that UGDH, as a dehydrogenase, converted UDP‐glucose (UDP‐Glc) to UDP‐glucuronic acid (UDP‐GlcA), which is a key precursor for the synthesis of glycosaminoglycan (GAGs).^[^ ^17^ ^]^ Intrigued by the previous findings, we set out to explore the effect of UGDH on GAGs synthesis by alcian blue staining and ELISA assays. Results showed that UGDH knockdown considerably diminished the efficacy of DCA in mitigating IL‐1β‐mediated GAGs loss (Figure 3L). To further investigate the effect of UGDH lactylation on GAGs synthesis, we measured GAGs production, UGDH activity, and concentrations of UDP‐Glc and UDP‐GlcA, respectively. Lactate treatment resulted in severe GAGs loss (Figure 3M) and notably inhibited UGDH activity (Figure 3N), leading to an increase in UDP‐Glc and a decrease in UDP‐GlcA (Figure 3N). Conversely, DCA relieved IL‐1β‐mediated GAGs loss (Figure 3O) and significantly enhanced UGDH activity (Figure 3P), which correspondingly decreased UDP‐Glc and increased UDP‐GlcA (**Figure** **4**P). Together, our results prove that UGDH lactylation aggravates ECM degradation and chondrocytes apoptosis in lactylation‐mediated OA.

### 2.5 K6 And K399 Residues are the Lactylated Lysine Sites in UGDH

To identify the lactylation sites of UGDH, we performed immunoprecipitation using an anti‐UGDH antibody, followed by LC‐MS analysis. The results showed that the K6 and K399 residues of UGDH were lactylated (Figure 4A). To validate these findings, we constructed flag‐tagged UGDH plasmids containing the wild type (WT), as well as K6R and K399R mutations, where lysine (K) at residue 6th and 399th were replaced by arginine (R). Subsequently, we conducted immunoprecipitation associated western blotting to assess the lactylation levels (Kla) of flag‐tagged UGDH. The mutation at the K6 site significantly reduced lactylation levels, whereas the K399 site mutation had no notable effect (Figure 4B). Notably, the K6 site and adjacent residues were highly conserved across multiple species (Figure 4C), unlike the K399 site, which exhibited less conservation (Figure S3, Supporting Information). This suggests that lactylation at K6 is crucial for UGDH function.

Overexpression of WT UGDH markedly decreased the expression of MMP3, MMP13, and ADAMTS4, while enhancing the synthesis of Collagen II and Aggrecan (Figure 4D,E). In comparison to the WT group, the K6R mutant exhibited lower expression levels of MMP3, MMP13, and ADAMTS4, along with increased synthesis of Collagen II and Aggrecan (Figure 4D,E). In contrast, the overexpression of K399R mutant did not significantly impact ECM degradation. In regulation of apoptosis, the K6R variant demonstrated decreased expression of cleaved‐caspase 3 and Bax, and increased expression of Bcl‐2, while the K399R group showed no difference with the WT group (Figure 4F). Consistently, overexpression of K6R UGDH significantly decreased the positive rate of apoptotic cells in immunofluorescence assay relative to the WT, whereas the K399R group did not differ from WT (Figure 4G).

For GAGs synthesis, K6R group showed higher GAGs synthesis than WT group, with no differences observed in the K399R group (Figure 4H). In addition, the K6R group showed increased UGDH activity, a decrease in UDP‐Glc, and an increase in UDP‐GlcA relative to the WT, while the K399R group exhibited no differences (Figure 4I). Collectively, our findings indicate that K6 lactylation is critical for UGDH lactylation and plays an essential role in ECM degradation and apoptosis in chondrocytes.

### 2.6 Lactylation of UGDH Facilitates its Nucleocytoplasmic Translocation

**Figure 5:**
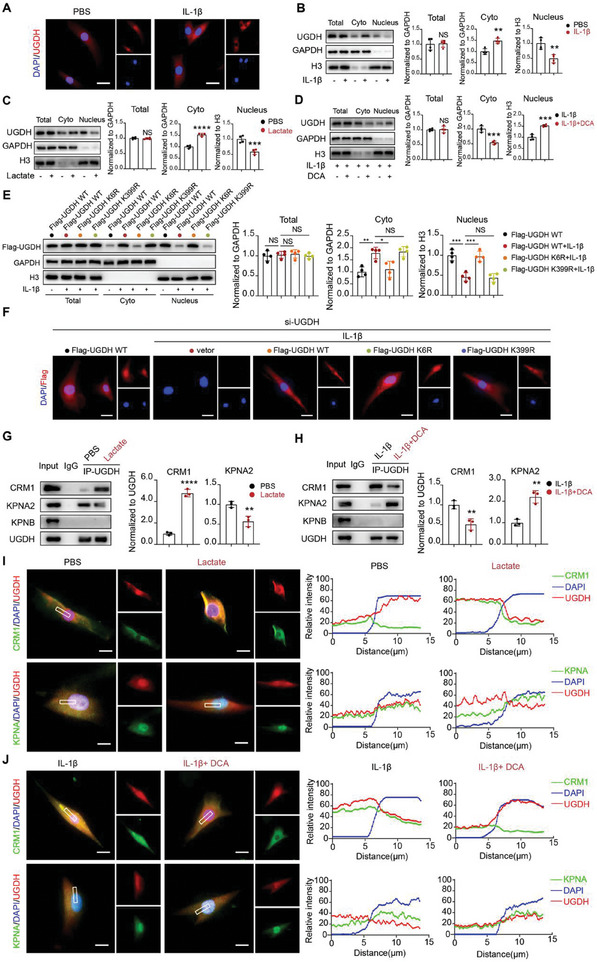
Lactylation of UGDH facilitates its nucleus to cytoplasm translocation. A) Representative immunofluorescence images of UGDH in the presence of IL‐1β (10 ng ml^−1^) or PBS. Scale bar, 25 µm. B–D) Western blot analysis of total UGDH, UGDH in cytoplasm (cyto) and nucleus under the stimulation of IL‐1β (10 ng ml^−1^) (**B**) or lactate (25 mm) (**C**) or DCA (20 mm) (**D**) and their relative control. Total UGDH and UGDH in cytoplasm were normalized to GAPDH. UGDH in nucleus was normalized to histone 3 (H3). E,F) Western blot analysis and representative immunofluorescence images of Flag‐UGDH WT, Flag‐UGDH K6R and Flag‐UGDH K399R in chondrocytes. Scale bar, 20 µm. G) Interaction assay of UGDH with CRM1, KPNA2 and KPNB in PBS or lactate (25 mm) treated chondrocytes. CoIP was conducted with Anti‐UGDH antibody. H) Interaction assay of UGDH with CRM1, KPNA2 and KPNB in IL‐1β (10 ng ml^−1^) or IL‐1β (10 ng ml^−1^) + DCA (20 mm) treated chondrocytes. CoIP was conducted with Anti‐UGDH antibody. I) Representative immunofluorescence images of UGDH (red) with CRM1 (green) or KPNA2 (green) in PBS or lactate (25 mm) treated chondrocytes. Scale bar, 20 µm (*n* = 4). J) Representative immunofluorescence images of UGDH (red) with CRM1 (green) or KPNA2 (green) in IL‐1β (10 ng ml^−1^) or IL‐1β (10 ng ml^−1^) + DCA (20 mm) treated chondrocytes. Scale bar, 20 µm. All data were presented as mean ± SD and analyzed by Student's t‐test. N represents the number of independent repeated experiments. NS: no significance, *P < 0.05, **P < 0.01, ***P < 0.001, and ****P < 0.0001.

Under physiological conditions, UGDH is distributed in both the nucleus and cytoplasm. Upon IL‐1β stimulation, UGDH translocates from the nucleus to the cytoplasm (**Figure** **5**A). To verify this translocation, UGDH was separately extracted from nuclear and cytoplasmic compartments. In the presence of IL‐1β and lactate, the total cellular UGDH levels remained constant, whereas its nuclear levels decreased and cytoplasmic levels increased (Figure 5B,C). DCA could attenuate the nuclear‐to‐cytoplasmic translocation caused by IL‐1β (Figure 5D). Under IL‐1β stimulation, wild‐type (WT) UGDH and K399R mutant translocated from the nucleus to the cytoplasm, while the translocation was attenuated in K6R mutant group (Figure 5E). Similarly in fluorescence experiments, WT UGDH and K399R mutant were primarily located in cytoplasm, while K6R mutant was mainly distributed in nucleus (Figure 5F). These results indicated that K6 lactylation facilitated UGDH translocation from the nucleus to cytoplasm. MS analysis indicated potential interactions between UGDH and several nuclear transport receptors, including karyopherin subunit alpha 2 (KPNA2), karyopherin beta (KPNB) and the nuclear export receptor chromosome region maintenance 1 (CRM1). Further investigation using CoIP assays showed that lactate enhanced the UGDH‐CRM1 interaction and diminished UGDH‐KPNA2 binding (Figure 5G). With IL‐1β stimulation, DCA reduced UGDH‐CRM1 interaction while increasing UGDH‐KPNA2 binding (Figure 5H). No interaction between UGDH and KPNB was observed (Figure 5G,H). Consistent with these findings, immunofluorescence assays demonstrated strong colocalization of UGDH with CRM1, and weak colocalization with KPNA2 in the presence of lactate (Figure 5I). However, under DCA stimulation, strong colocalization of UGDH with KPNA2 and weak colocalization with CRM1 were detected (Figure 5J). Collectively, UGDH lactylation facilitates its nucleus‐to‐cytoplasm translocation by regulating its interaction with CRM1 and KPNA2.

### 2.7 Lactylation Of UGDH Inhibits its Interaction with STAT1 and Promotes STAT1‐Mediated MAP3K8 Transcription

**Figure 6:**
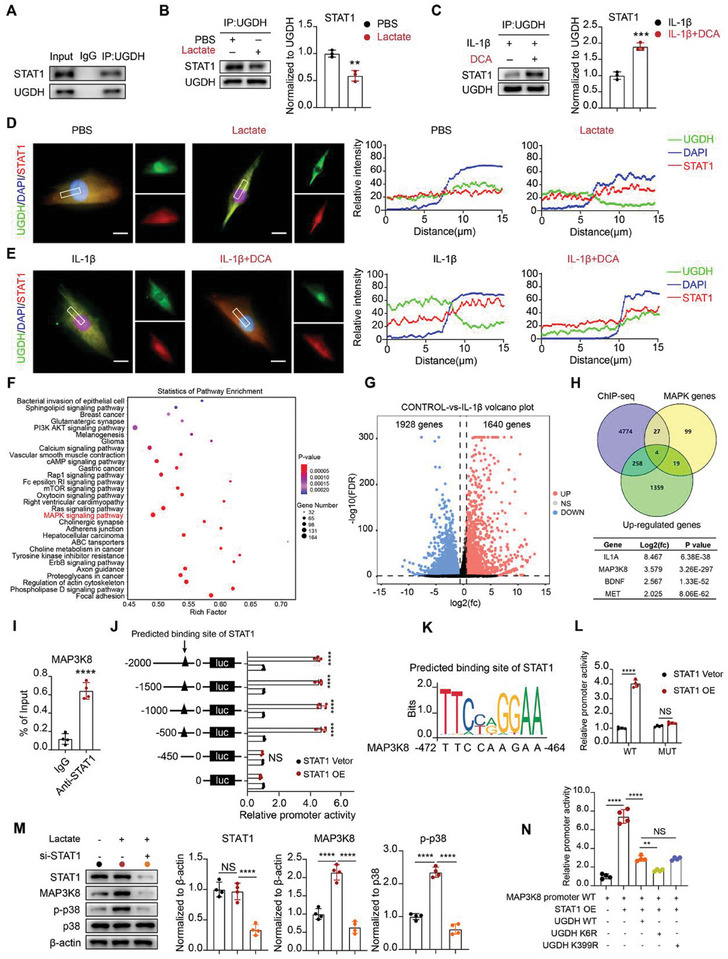
Lactylation of UGDH inhibits its interaction with STAT1 and promotes STAT1 mediated MAP3K8 transcription. A) CoIP of STAT1 by UGDH antibody. B) Interaction assay of UGDH with STAT1 in PBS or lactate (25 mm) treated chondrocytes. CoIP was conducted with Anti‐UGDH antibody. The data were normalized to UGDH. C) Interaction assay of UGDH with STAT1 in IL‐1β (10 ng ml^−1^) or IL‐1β (10 ng ml^−1^) + DCA (20 mm) treated chondrocytes. CoIP was conducted with Anti‐UGDH antibody. The data were normalized to UGDH. D) Representative immunofluorescence images of UGDH (green) with STAT1 (red) in PBS or lactate (25 mm) treated chondrocytes. Scale bar, 20 µm. E) Representative immunofluorescence images of UGDH (green) with STAT1 (red) in IL‐1β (10 ng ml^−1^) or IL‐1β (10 ng ml^−1^) + DCA (20 mm) treated chondrocytes. Scale bar, 20 µm. F) Statistics of pathway enrichment. Data from CHIP‐seq with anti‐STAT1 antibody. G) Volcano plot of a transcriptome profiling dataset involving ten IL‐1β treated human chondrocytes and its negative controls (GSE162510). H) Venn plot of the genes of CHIP‐seq, MAPK pathway related genes and upregulated genes in IL‐1β treated chondrocytes and its log2(fc) and p value. I) Relative quantification of ChIP‐qPCR for MAP3K8. J) Binding region prediction assay of STAT1 at MAP3K8 promoter. HEK293T cells were transfected with luciferase reporter gene (luc) labeled plasmids containing truncated MAP3K8 promoter and treated with STAT1 vetor (control) or STAT1 plasmids (STAT1 OE) for 24 h, followed by luciferase reporter assays. K) Predicted binding site of STAT1 at MAP3K8 promoter by JASPAR online tools. L) Luciferase reporter assays. HEK293T cells were transfected with luciferase reporter gene labeled plasmids containing WT MAP3K8 promoter (WT) or mutated MAP3K8 promoter (MUT) under the treatment of STAT1 vetor (control) or STAT1 plasmids (STAT1 OE) for 24 h. M) Western blot of STAT1, MAP3K8 and p‐p38 in chondrocytes with PBS (control group) or lactate (25 mm) or lactate (25 mm) + si‐STAT1 (STAT1 siRNA) treatment (*n* = 4). **N** HEK293T cells were transfected with UGDH WT, UGDH K6R, UGDH K399R plasmids under the treatment of STAT1 plasmids and luciferase reporter gene labeled MAP3K8 WT plasmids, followed by luciferase reporter assays. The data were normalized to β‐actin. All data were presented as mean ± SD and analyzed by Student's t‐test or One‐way analysis of variance (ANOVA) with Dunnett's post hoc test. N represents the number of independent repeated experiments. NS: no significance, *P < 0.05, **P < 0.01, ***P < 0.001, and ****P < 0.0001.

To investigate the specific mechanism of UGDH in OA, UGDH was purified by CoIP assays followed by MS analysis. According to the MS analysis, a series of UGDH binding peptides were detected with significant enrichment of STAT1‐specific peptides. STAT1, a well‐known transcription factor, participates in a variety of biological processes.^[^ ^18^ ^]^ To further verify the interaction of UGDH and STAT1, CoIP with an anti‐UGDH antibody confirmed the interaction between UGDH and STAT1 (**Figure** **6**A). In addition, lactate weakened the interaction between UGDH and STAT1 (Figure 6B), while DCA, under IL‐1β stimulation, strengthened their interaction (Figure 6C). Immunofluorescence assays showed reduced colocalization of UGDH and STAT1 in lactate‐treated chondrocytes (Figure 6D), but strong colocalization under DCA treatment was noted (Figure 6E). K6 mutation of UGDH facilitated the combination of UGDH and STAT1. However, compared with WT UGDH, K399 mutation of UGDH showed no difference in the combination of UGDH and STAT1(Figure S4A, Supporting Information).

To investigate the role of STAT1 as a transcription factor, chromatin immunoprecipitation sequencing (ChIP‐seq) with an anti‐STAT1 antibody was performed. Pathway enrichment analysis revealed that MAPK pathway genes were highly enriched by STAT1 (Figure 6F). Additionally, phosphorylated p38 (p‐p38) levels increased in lactate‐treated chondrocytes and decreased in DCA‐treated ones (Figure S4B,C, Supporting Information), implicating the p38‐MAPK pathway in lactylation‐driven OA progression. To fully elucidate the mediator between STAT1 and p38‐MAPK pathway, we analyzed a published transcriptome profiling dataset involving ten IL‐1β‐treated human chondrocytes samples and its negative controls (GSE162510).^[^ ^19^ ^]^ Volcano plot indicated that 1640 genes were significantly upregulated in IL‐1β treated chondrocytes (Figure 6G). Further overlapped the genes of CHIP‐seq, MAPK pathway related genes and upregulated genes in IL‐1β treated chondrocytes, we identified four candidate genes for the mediator between STAT1 and p38‐MAPK pathway (Figure 6H), including interleukin‐1 alpha (IL1A), MAP3K8, brain derived neurotrophic factor (BDNF) and MET proto‐oncogene receptor tyrosine kinase (MET). Among them, only IL1A and MAP3K8 mRNAs were significantly upregulated with lactate treatment and downregulated with DCA treatment (Figure S4D,E, Supporting Information).

Next, we tested whether STAT1 could bind to the promoters of IL1A and MAP3K8. ChIP‐qPCR confirmed STAT1 binding at the MAP3K8 promoter but not IL1A (Figure 6I; Figure S4F, Supporting Information). To map the STAT1 binding region on MAP3K8 gene, we used luciferase reporter plasmids containing sequences of various lengths ranging from −2000 bp upstream of the transcription start site (TSS) to the 5′ flanking region of MAP3K8. The results showed that plasmids containing sequences from −450 bp to TSS did not affect luciferase activity induced by STAT1 overexpression, whereas plasmids containing sequences from −500 bp or longer to TSS significantly enhanced STAT1 overexpression‐induced luciferase activity (Figure 6J). These results indicate that the STAT1 binding region on the MAP3K8 promoter is located between −2000 and −450 bp upstream of the TSS. Using the JASPAR online tool, the specific binding site of STAT1 at the promoter of MAP3K8 was predicted (Figure 6K). Mutation of the predicted STAT1‐binding site resulted in a significant reduction in luciferase activity induced by STAT1 overexpression (Figure 6L). Consistent with these findings, lactate promoted the expression of MAP3K8 and p‐p38 but not STAT1 (Figure 6M). Knockdown of STAT1 significantly reduced the expression of MAP3K8 and p‐p38 (Figure 6M).

To assess the effects of UGDH lactylation on STAT1‐mediated transcription of MAP3K8, HEK293T cells were transfected with UGDH WT, UGDH K6R, UGDH K399R plasmids along with STAT1 plasmids and luciferase reporter gene‐labeled MAP3K8 WT plasmids. Results showed that UGDH WT group significantly inhibited STAT1‐mediated transcription of MAP3K8. Furthermore, compared to the UGDH WT group, the K6R group exhibited stronger repression of STAT1‐mediated transcription of MAP3K8, while the K399R group showed no significant difference (Figure 6N). These results indicate that the lactylation of UGDH hampers its interaction with STAT1, subsequently enhancing STAT1‐mediated transcription of MAP3K8, ultimately leading to the activation of p38‐MAPK pathway.

### 2.8 P300 Inhibitor A485 Rescues OA Progression In Vivo and In Vitro

**Figure 7:**
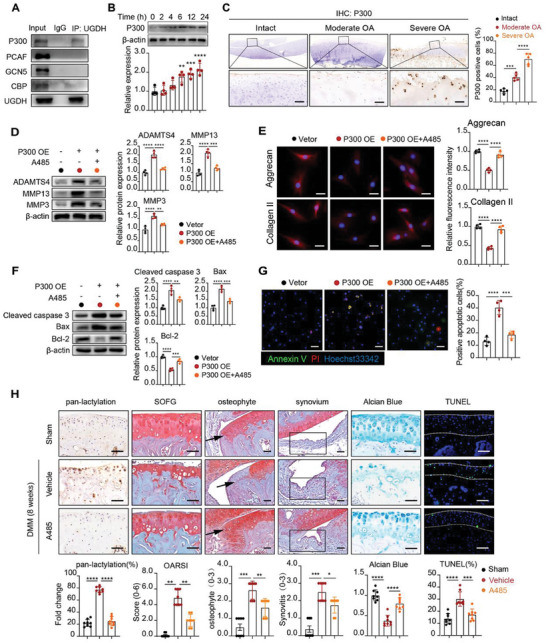
P300 inhibitor A485 rescues OA progression in vivo and in vitro. A) CoIP of UGDH with Anti‐UGDH antibody, follow by Western blot analysis of P300, PCAF, GCN5, and CBP. B) Western blot of P300 in chondrocytes with IL‐1β (10 ng ml^−1^) treatment for 0, 2, 4, 6, 12, and 24 h. The data were normalized to β‐actin. C) Immunohistochemistry (IHC) of P300 in the cartilage from OA patients (5 samples per group). Scale bars, 50 µm. D) Western blot analysis of MMP3, MMP13 and ADAMTS4 expression in chondrocytes transfected with P300 overexpression (OE) and treated with A485. The data were normalized to β‐actin (*n* = 4). E) Immunofluorescence staining of Aggrecan and Collagen II in the same conditions as in D (*n* = 4). Scale bar, 25 µm. F) Western blot analysis of cleaved‐caspase 3, Bax, and Bcl‐2 expression with quantification normalized to β‐actin (*n* = 4). G) Representative immunofluorescence images of Annexin V (green) and PI (red) in chondrocytes, with quantification of apoptotic cell percentage (*n* = 4). Scale bar, 100 µm. H) IHC of pan‐lactylation of articular cartilage in sham, DMM induced OA mice with A485 or DMSO. (8 mice per group). Scale bars, 50 µm. SOFG of articular cartilage, osteophyte (arrow mark) and synovium (rectangle mark) in sham, DMM induced OA mice with A485 or DMSO. OARSI grade was measured according to SOFG staining. Scale bars, 50 µm. Osteophyte score for analysis of osteophyte formation. Scale bars, 100 µm. Synovium score for analysis of synovitis. Scale bars, 100 µm. Alcian blue staining of articular cartilage in sham, DMM induced OA mice with A485 or DMSO. Scale bars, 50 µm. TUNEL analysis of articular cartilage in sham, DMM induced OA mice with A485 or DMSO. Green fluorescence represented TUNEL positive cells. Blue fluorescence (DAPI) represented all cells. Scale bars, 100 µm. Data were presented as mean ± SD and analyzed by One‐way analysis of variance (ANOVA) with Dunnett's post hoc test. Scores of OARSI, osteophyte and synovitis were shown as mean ± 95% CI and Mann‐Whitney U test for statistical analysis. N represents the number of independent repeated experiments or sample size. NS: no significance, *P < 0.05, **P < 0.01, ***P < 0.001, and ****P < 0.0001.

To identify the potential catalytic enzymes responsible for UGDH lactylation, we examined its potential interaction with a range of common acyltransferases, including P300, PCAF, GCN5 and CBP.^[^ ^14^ ^]^ The results showed that P300 was the only enzyme specifically interacting with UGDH (**Figure** **7**A). P300 is an important acetyltransferase as well as a key regulatory molecule in lactylation, acting as a “writer” that adds lactate groups to lysine residues.^[^ ^20^ ^]^ Upon IL‐1β stimulation, the level of P300 showed a time‐dependent increase (Figure 7B). Compared to intact cartilage, P300 expression increased progressively with cartilage degradation severity in both humans (Figure 7C) and mice (Figure S5A, Supporting Information), indicating that P300 might play a role in OA progression.

To investigate the roles of P300 on ECM degradation and chondrocyte apoptosis, we conducted P300 overexpression (OE) plasmid transfection and treatments with A485, a selective P300 inhibitor.^[^ ^21^ ^]^ Western blot analysis revealed that P300 overexpression significantly increased the expression of MMP3, MMP13, and ADAMTS4, along with decreased synthesis of Collagen II and Aggrecan, which was notably reduced by A485 treatment (Figure 7D,E). Additionally, P300 overexpression led to increased levels of cleaved‐caspase 3 and Bax, and a decrease in Bcl‐2, while A485 reversed these effects (Figure 7F). Annexin V/PI staining confirmed that P300 overexpression increased the percentage of apoptotic cells. Similarly, this effect was significantly reduced by A485 treatment (Figure 7G). These results suggest that P300 overexpression promotes ECM degradation and chondrocyte apoptosis and that A485 treatment effectively inhibits these effects.

Next, we investigated how UGDH mutations affect ECM degradation and chondrocyte apoptosis in response to P300 overexpression. The results showed that P300 overexpression significantly increased UGDH Kla expression in UGDH WT and K399R group, but this effect was reduced in UGDH K6R group (Figure S5B, Supporting Information). Further western blot analysis revealed that P300 overexpression significantly increased the expression of MMP3, MMP13, and ADAMTS4 in UGDH WT group, along with decreased synthesis of Collagen II and Aggrecan, while this effect was diminished in UGDH K6R group, but not K399R group (Figure S5C,D, Supporting Information). In regulation of apoptosis, P300 overexpression increased cleaved‐caspase 3 and Bax levels and decreased Bcl‐2 expression in UGDH WT group, while this effect was attenuated in UGDH K6R group, but not K399R group (Figure S5E, Supporting Information). Annexin V/PI staining further confirmed that P300 overexpression increased the percentage of apoptotic cells in UGDH WT group, this response was significantly reduced in UGDH K6R group (Figure S5F, Supporting Information). These results suggest that UGDH K6R mutation relieve ECM degradation and chondrocyte apoptosis induced by P300 overexpression.

Further, we detected the effect of A485 on UGDH lactylation‐mediated OA progression. The results showed that A485 treatment significantly inhibited UGDH Kla (Figure S6A, Supporting Information). As expected, administration of A485 notably reduced the synthesis of MMP3, MMP13, and ADAMTS4, while enhancing the expression of Collagen II and Aggrecan in the presence of IL‐1β (Figure S6B,C, Supporting Information). For apoptosis detection, A485 inhibited the expressions of cleaved‐caspase 3 and Bax, while promoting Bcl‐2 expression (Figure S6D, Supporting Information). In line with these observations, A485 reduced the proportion of apoptotic cells revealed by immunofluorescence assays (Figure S6E, Supporting Information). For GAGs synthesis, A485 treatment significantly increased GAGs production (Figure S6F, Supporting Information). In addition, UGDH activity was enhanced in the A485‐treated group, accompanied by a decrease in UDP‐Glc and an increase in UDP‐GlcA (Figure S6G, Supporting Information).

For in vivo analysis of A485 functions, we conducted articular injection in DMM‐induced OA mice, treating with A485 (with DMSO as control) for 8 weeks (Figure S7A, Supporting Information). IHC results demonstrated that A485 treatment significantly reduced cartilage pan‐lactylation levels (Figure 7H). SOFG staining results showed that A485 treatment significantly alleviated cartilage loss, osteophyte formation and synovitis in DMM‐induced OA (Figure 7H). Alcian blue staining showed that A485 treatment effectively mitigated GAGs loss in DMM‐induced OA (Figure 7H). For apoptosis in vivo, A485 treatment markedly decreased the rate of green fluorescence‐labeled apoptotic cells within cartilage (Figure 7H). In addition, IHC results demonstrated that A485 treatment significantly increased the expressions of Collagen II and Aggrecan, while reducing the expressions of MMP3 and MMP13 in DMM‐induced OA (Figure S7B, Supporting Information). Taken together, our data suggest that inhibiting P300 by A485 could alleviate chondrocyte degradation and OA progression.

## 3 Discussion

In this study, we uncovered the elevated lactylation levels in OA and its detrimental role in disease progression. We further identified that K6 lactylation of UGDH weakened the protective effects of UGDH by reducing GAGs synthesis and regulating its nuclear‐cytoplasmic distribution. Therapeutically, inhibition of P300 by its specific inhibitor A485 significantly reduced UGDH lactylation and alleviated OA progression. Mechanically, we proved that K6 lactylation of UGDH repressed its enzymatic activity, and impeded the interaction of UGDH with STAT1 in nucleus, thus promoted the transcription of MAP3K8 and activated MAPK pathway. Our findings reveal the mechanism of lactylation and provide a new therapeutic target for OA.

The progression of OA has been closely linked to alterations in metabolic pathways, particularly dysregulated glycolysis.^[^ ^22^, ^23^ ^]^ Glycolysis, the primary source of energy in chondrocytes, is perturbed in OA, leading to accumulation of lactate.^[^ ^24^ ^]^ Elevated lactate levels have been implicated in promoting inflammatory responses and cartilage degradation.^[^ ^25^ ^]^ Our study identified increased lactate synthesis in synovial fluid, which was consistent with a previous study.^[^ ^11^ ^]^ However, the source of lactate in synovial fluid has rarely been studied. We proved that the expressions of main rate‐limiting enzymes in glycolysis, including HK2, PFPL, PFKP, and PKM were elevated in various cells of OA joint tissue, especially in synovial fibroblast. Meanwhile, LDHA, the key enzyme for lactate synthesis, also showed an overall increasing trend. We validated that metabolic reprogramming occurred in multiple joint tissues during the onset of OA, not just in cartilage, highlighting the need for further exploration of lactate's multifaceted roles in OA pathogenesis. Lactylation, a PTM of proteins driven by lactate, has surfaced as a novel regulatory mechanism in biological processes.^[^ ^12^ ^]^ Though lactylation has been associated with various diseases such as cancer and neurological disorders,^[^ ^26^, ^27^ ^]^ its role in OA remains unknown. Current researches on lactate in OA primarily focuses on its role as a metabolic waste product or signaling molecule without delving into PTM it may induce.^[^ ^28^ ^]^ Our study reported the global lactylation levels in OA cartilage, demonstrating a significant increase compared to healthy controls in vivo and in vitro. Furthermore, both in vivo and in vitro studies showed that regulating the levels of lactylation could effectively modulate ECM homeostasis and apoptosis in chondrocytes. This finding suggested that lactylation may represent a previously unrecognized mechanism by which lactate exerts its deleterious effects in OA, opening up new avenues for investigation into the PTM contributing to disease progression.

Currently, most researches on lactylation focus on histone lactylation, where lactylation exerts its effects by influencing the transcriptional regulation of target genes by histones.^[^ ^29^ ^]^ However, there are limited researches on non‐histone lactylation. Actually, lactylation can also directly regulate the structure or stability of non‐histone proteins to exert its functions.^[^ ^30^ ^]^ In our current study, UGDH was demonstrated to be lactylated in chondrocytes. UGDH is a critical enzyme involved in the synthesis of GAGs, a major component of the ECM that maintains cartilage integrity.^[^ ^31^ ^]^ Reduced UGDH has been associated with cartilage degeneration in OA.^[^ ^32^ ^]^ Activation of UGDH by transforming growth factor β (TGF‐β) can promote the synthesis of GAGs in articular chondrocytes and relieve OA progression.^[^ ^33^ ^]^ However, the specific mechanism of UGDH in OA remains unknown. In this study, we found that knockdown of UGDH with UGDH siRNA significantly decreased the synthesis of GAGs, which was consistent with a previous study.^[^ ^31^ ^]^ Surprisingly, under the stimulation of IL‐1β, the expression of UGDH did not change significantly while the lactylation level of UGDH increased. Further, we proved that elevated lactylation of UGDH accelerated OA by inhibiting GAGs synthesis, promoting ECM catabolism and chondrocyte apoptosis. As a dehydrogenase, the enzymatic activity of UGDH was significantly repressed by its lactylation, thus blocked the conversion of UDP‐Glc to UDP‐GlcA, ultimately led to the decrease of GAGs. These results indicate that UGDH is the key factor of lactylation mediated OA.

In addition, our study revealed a deeper aspect of UGDH regulation through K6 lactylation, which weakened its protective effects by altering its subcellular localization. As a functional protein regulated by lactylation in OA, UGDH was found to translocate from the nucleus to the cytoplasm after lactylation. The above results indicated that the subcellular localization of UGDH affected its role in OA. Like most macromolecular proteins, the nucleocytoplasmic transport of UGDH was achieved by directly binding to nuclear transport proteins.^[^ ^34^ ^]^ Differently, the nucleocytoplasmic transport of UGDH was regulated by K6 lactylation. As described above, in cytoplasm, the enzymatic activity of UGDH has been inhibited by lactylation. However, the roles of UGDH in nucleus are still unknown. Further, our study uncovered an interplay between UGDH and the transcription factor STAT1 in nucleus. STAT1, traditionally recognized for its role in transducing signals from the cell membrane to the nucleus, is implicated in a distinct regulatory mechanism within the context of OA.^[^ ^35^, ^36^ ^]^ Specifically, we demonstrated that UGDH lactylation disrupted its interaction with STAT1 in the nucleus. This disruption had profound implications, as it promoted the transcription of MAP3K8 and subsequent activation of the MAPK pathway. Notably, MAP3K8 stands out as a key upstream activator of the MAPK pathway and is highly expressed in OA articular cartilage.^[^ ^37^ ^]^ MAPK pathway has a pivotal role in OA, serving as a central mediator of inflammatory responses and catabolic processes that ultimately contribute to cartilage degradation.^[^ ^38^ ^]^ This intricate signaling network orchestrates a multitude of cellular activities, including proliferation, differentiation, and apoptosis, all of which are dysregulated in OA chondrocytes. The disruption of the UGDH‐STAT1 interaction by lactylation highlighted a previously unrecognized layer of complexity in OA pathogenesis. This finding suggested that PTM could significantly impact transcription factor function and signaling pathway activation, ultimately influencing disease progression. By elucidating this novel regulatory mechanism, we gain new insights into the complex regulatory networks that govern OA and identify potential targets for therapeutic intervention.

P300 is involved in OA progression as the transcription activator of inflammatory factors.^[^ ^39^ ^]^ It promotes the transcription of target gene by enhancing the level of histone acetylation.^[^ ^40^ ^]^ In addition, P300 is a key regulatory molecule in lactylation of protein.^[^ ^20^ ^]^ However, the role of P300 as the writer of lactylation in OA has not been studied yet. Here, we identified that P300 could interact with UGDH. Inhibition of P300 by its specific inhibitor A485 significantly decreased UGDH lactylation levels and alleviated GAGs loss, ECM degradation and chondrocyte apoptosis in vivo and in vitro. As reported, A485 inhibits the activity of P300 by bonding with its catalytic active site.^[^ ^41^ ^]^ To our knowledge, the effect of A485 has not been tested in OA. These findings suggest that targeting this lactylation event by A485 could be a promising therapeutic strategy.

While our study investigates the roles of UGDH lactylation in OA progression, several limitations should be acknowledged. First, a UGDH(K6)‐specific lactylation antibody would be a valuable tool for further validation in vivo and in vitro. Also, the specific mechanism by which K6 lactylation precisely modulates the nuclear‐cytoplasmic shuttling of UGDH and interaction with STAT1 remains to be fully elucidated. This knowledge could lead to the development of novel therapeutic strategies targeting these interactions to treat or prevent OA progression associated with altered nuclear‐cytoplasmic transport. Second, OA is a disease that affects the entire joint. Currently, we have only explored the mechanism of lactylation in articular cartilage, and the effects of lactylation in other joint structures on OA are also worthy of attention, such as the synovium and infrapatellar fat pad. Last, the specificity and long‐term efficacy of the P300 inhibitor A485 in treating OA need to be rigorously tested in larger‐scale preclinical and clinical trials.Whether the protective effects of A485 on OA is solely achieved by inhibiting UGDH lactylation or there may also be the involvement of other pathways need further validation. Addressing these limitations will help refine our understanding of lactylation in OA and facilitate the development of effective therapeutic strategies.

In conclusion, our study sheds light on the previously unexplored role of lactylation in OA, identifying UGDH as a major target of this modification and elucidating its mechanistic implications in regulating GAGs synthesis and MAPK signaling. These findings not only advance our understanding of OA pathogenesis but also suggest potential therapeutic targets for this debilitating disease.

## 4 Experimental Section

### Ethics

This study was approved by the Ethics Committee of Zhujiang Hospital, Southern Medical University. All human tissue samples involved in this study were obtained with full informed consent from patients and in accordance with the approval of the Ethics Committee of Zhujiang Hospital, Southern Medical University. All animal experiments were strictly conducted in accordance with national and international ethical regulations, and are approved by the Ethics Committee of Zhujiang Hospital, Southern Medical University (LAEC‐2022‐150).

### Human Primary Chondrocytes Isolation and Culture

Primary chondrocytes were isolated as previously described.^[^ ^42^ ^]^ Under sterile conditions, human cartilage tissues were washed three times with phosphate buffered saline (PBS), and then cut into small pieces (0.5 mm^3^). The cartilage was digested with trypsin (Gibco,25300‐054) at three times its volume for 30 min (min) at 37 °C. Subsequently, centrifugation was performed at 40 g for 5 min, and the supernatant was removed. The cartilage fragments were then rinsed with sterile PBS, followed by centrifugation at 40 g for 5 min and removal of the PBS. After that, digestion was carried out using five times volumes of 0.2% type II collagenase in a 37 °C constant temperature oscillator for 16 h. The suspension was centrifuged at 40 g for 5 min to separate the supernatant and undigested tissue precipitate. Five times volumes of 0.2% type II collagenase (Gibco,17 101 015) were added to the undigested tissue precipitate for another 16 h of digestion to obtain more cells. The supernatant was then centrifuged at 300 g for 5 min, and the cell precipitate was retained. The cell precipitate was resuspended in 5 ml of DMEM/F12 (Gibco,11 320 033) medium containing 10% fetal bovine serum (Gibco,10099‐141) and cultured in a 37 °C humidified atmosphere with 5% CO_2_. The culture medium was changed every three days. When reaching 80% confluence, primary chondrocytes could be passaged or used for further cell experiments.

### Animal Experiments

All animal experiments were strictly conducted in accordance with national and international ethical regulations, and were approved by the Ethics Committee of Zhujiang Hospital, Southern Medical University (LAEC‐2022‐150). Ten‐week‐old male C57BL/6 mice were randomly assigned to each experimental group (*n* = 8). The experimental OA models were induced by destabilization of the medial meniscus (DMM) surgery as previously reported.^[^ ^43^ ^]^ After anesthesia with isoflurane, the right knee joint was exposed by skin incision under the stereomicroscope. Then the medial meniscus was destabilized by resection of medial meniscotibial ligaments. Finally, hemostasis was performed and the skin was sutured. Safranin O and Fast Green staining (SOFG) was performed at 2, 4, and 8 weeks (W) after surgery to detect the degree of cartilage damage. According to the experimental design, lactate group: non‐surgical mice received articular injections of 10 µL Lactate (25 mm, MedChemExpress, HY‐B2227B) at 0, 2, 4, and 6 W, articular injections of 10 µL phosphate buffered saline (PBS) as control; DCA group: DMM mice received articular injections of 10 µL DCA (20 mm, MedChemExpress, HY‐Y0445A) at 1, 3, 5, and 7 W, articular injections of 10 µL PBS as control; A485 group: DMM mice received articular injections of 2 µL A485 (20 mm, MedChemExpress, HY‐107455) at 1, 3, 5, and 7 W, articular injections of 2 µL Dimethyl sulfoxide (DMSO) as control. All mice were sacrificed at 8 W after DMM surgery or the first articular injection.

### Analysis of Differential Expression of Genes Related to Glycolysis

As described in the previous study,^[^ ^15^ ^]^ raw single‐cell RNA sequencing (scRNA‐seq) data of normal and OA cartilage tissues were retrieved from the Gene Expression Omnibus (GEO) database under accession numbers GSE169454. Additionally, raw single‐nucleus RNA sequencing (snRNA‐seq) files pertaining to single‐nucleus human perivascular adipose tissue were obtained from GEO, specifically from GSE164528 and GSE176171. The glycolysis/gluconeogenesis‐specific KEGG pathway annotation (hsa00010) was employed as a framework to construct heatmaps, which facilitated the visualization of metabolic dysregulations observed in both normal and OA joint tissues.

### Lactate Detection

The lactate concentration of human synovial fluid and chondrocytes culture medium was measured as manufacture's instruction by using CheKine Lactate Assay Kit (Abbkine, China, KTB1100). Synovial fluid was collected from non‐OA donors and OA patients (Table S1, Supporting Information). Chondrocytes were treated with 10 ng ml^−1^ interleukin‐1β (IL‐1β, Sigma‐Aldrich, St. Louis, Mo, USA) for 0, 2, 4, 6, 12, and 24 h to construct an inflammatory chondrocyte model, then culture medium was collected for lactate measurement.

### Regulation of Lactylation Level In Vitro

Chondrocytes were seeded in 6‐well plates. When reaching 80% confluence, chondrocytes were treated with Lactate (25 mm, MedChemExpress, HY‐B2227B) to increase its lactylation level. On the contrary, Dichloroacetic acid (DCA, 20 mm, MedChemExpress, HY‐Y0445A) or P300 inhibitor A485 (5, 10, 20 mm, MedChemExpress, HY‐107455) were added to decrease the lactylation level of chondrocytes under the treatment of IL‐1β (Sigma‐Aldrich, St. Louis, Mo, USA). After 24 h of stimulation, Chondrocytes were harvested for further experiments.

### Histological Analysis, Immunohistochemistry (IHC) and TUNEL Analysis

Human cartilage tissues and mouse joints were fixed in 4% paraformaldehyde (Biosharp, BL539A), decalcified in 0.5 µm EDTA, embedded in paraffin, and sectioned into 4 um slices. All slides were deparaffinized in xylene, rehydrated with graded ethanol, and stained with 0.2% Safranin O and Fast Green (Sigma‐Aldrich, USA). OARSI grade was measured according to SOFG staining.^[^ ^44^ ^]^ Osteophyte score was assessed for analysis of osteophyte formation.^[^ ^44^ ^]^ Synovium score was assessed for analysis of synovitis.^[^ ^45^ ^]^ For immunohistochemistry, the slides were treated with EDTA solution in a boiling water bath for antigen retrieval. 5% bovine serum albumin (BSA, MedChemExpress, HY‐D0842) was used for blocking for 1 h. After that, the slides were incubated with primary antibodies (1:100) at 4 °C for 12 h (Anti‐Collagen II antibody, Abcam, ab34712; Anti‐aggrecan antibody, Abcam, ab3778; Anti‐MMP3 antibody, Proteintech, 17873‐1‐AP, Anti‐MMP13 antibody, Proteintech, 18165‐1‐AP; Anti‐Lactylation antibody, PTM BIO, PTM‐1401RM; Anti‐P300 antibody, Proteintech, 20695‐1‐AP). After incubation, the slides were washed three times with PBS and incubated with biotin‐conjugated goat anti‐rabbit secondary antibody (1:200, Proteintech, SA00001‐2) at room temperature for 2 h. After washing three times with PBS, the slides were stained with DAB (Thermo Scientific, 34 002) and counter‐stained with hematoxylin. For TUNEL analysis, after dewaxing and rehydration, the slides were incubated in 20 µg ml^−1^ of proteinase K (Beyotime, St532) at 37 °C for 30 min. 50 µl of TUNEL assay solution (Beyotime, C1088) was added to each sample and incubated at 37 °C in the dark for 60 min. Nuclei were stained with 4,6‐diamidino‐2‐phenylindole (DAPI, Solarbio, C0065). Nikon Ti2‐E was used to capture the fluorescence images.

### Immunofluorescence Staining

Chondrocytes were seeded in confocal dishes and treated with relative stimulus. After stimulation, chondrocytes were washed with PBS and fixed with 4% paraformaldehyde for 15 min. Chondrocytes were permeabilized by 1% Triton X‐100 for 10 min at room temperature and then blocked by 5% BSA for 30 min. Primary antibodies (1:200) (Anti‐ Collagen II antibody, Abcam, ab34712; Anti‐aggrecan antibody, Abcam, ab3778; Anti‐Lactylation antibody, PTM BIO, PTM‐1401RM; Anti‐UGDH antibody, Sino Biologica, 200912‐T44; Anti‐UGDH antibody, Proteintech, 67360‐1‐IG; Anti‐Flag antibody, Proteintech, 66008‐4‐Ig; Anti‐STAT1 antibody, Proteintech, 10144‐2‐AP; Anti‐CRM1 antibody, Proteintech, 66763‐1‐Ig; Anti‐KPNA2 antibody, HUABIO, EM1707‐61) were used for incubation at 4 °C for 12 h. Goat Anti‐Rabbit IgG H&L (Cy5) (1:500, abcam, ab6564) and Goat Anti‐Mouse IgG H&L (FITC) (1:500, abcam, ab6785) were used to incubate in the dark at room temperature for 1 h. Nuclei were stained with DAPI (Solarbio, C0065). Nikon Ti2‐E was used to capture the fluorescence images.

### Western Blot

After relative stimulation, cells were harvested and washed with PBS. Proteins were extracted with RIPA buffer (Beyotime, P0013B) containing 1% PMSF (Beyotime, ST505). Proteins were subjected to sodium dodecyl sulfate polyacrylamide gel electrophoresis (SDS‐PAGE) and transferred to polyvinylidene fluoride membranes (Millipore, C3117). Membranes were blocked with 5% non‐fat milk at room temperature for 1 h. Primary antibodies (1:1000) (Anti‐MMP3 antibody, Proteintech, 17873‐1‐AP, Anti‐MMP13 antibody, Proteintech, 18165‐1‐AP; Anti‐Lactylation antibody, PTM BIO, PTM‐1401; Anti‐ADAMTS4 antibody, Proteintech, 11865‐1‐AP; Anti‐P300 antibody, Proteintech, 20695‐1‐AP; Anti‐Bcl2 antibody, Proteintech, 68103‐1‐Ig; Anti‐Cleaved Caspase 3 antibody, Affinity Biosciences, AF7022; Anti‐Bax antibody, Proteintech, 50599‐2‐Ig; Anti‐UGDH antibody, Sino Biologica, 200912‐T44; Anti‐STAT1 antibody, Proteintech, 10144‐2‐AP; Anti‐CRM1 antibody, Proteintech, 66763‐1‐Ig; Anti‐KPNA2 antibody, HUABIO, EM1707‐61; Anti‐KPNB antibody, HUABIO, HA721702; Anti‐PCAF antibody, HUABIO, ET7106‐79; Anti‐GCN5 antibody, ABM, A2224; Anti‐CBP antibody, HUABIO, ER62918) and internal control antibodies (1:5000)(Anti‐β‐actin antibody, Proteintech, 81115‐1‐RR; Anti‐Flag antibody, Proteintech, 66008‐4‐Ig; Anti‐GAPDH antibody, Proteintech, 60004‐1‐Ig; Anti‐Histone H3 antibody, Proteintech, 17168‐1‐AP) were used for incubation at 4 °C for 12 h. After three washes with Tris Buffered Saline Tween (TBST), the membranes were incubated with horseradish peroxidase (HRP)‐conjugated anti‐rabbit IgG (1:5000, Boster, BA1054) or anti‐mouse IgG (1:5000, Boster, BA1050) at room temperature for 1 h. ECL (Millipore, WBKLS0100) and a chemiluminescence system (Bio‐Rad, USA) were used for chemiluminescent imaging. ImageJ software was used for the analyses.

### Apoptosis Detection

Apoptosis of chondrocytes was detected with Annexin V‐FITC apoptosis detection kit (Beyotime, C1062 M). As manufacturer's instructions, culture medium was removed. Mixture of staining solution containing 5 µl Annexin V‐FITC and 10 µl Propidium Iodide (PI) was added to each well and incubated at 37 °C for 30 min. Hoechst33342 (MCE, HY‐15559) was used to stain the nuclei. Nikon Ti2‐E was used to capture the fluorescence images. Annexin V‐FITC (green), PI (red) and hoechst33342 (blue) represented apoptotic cells, necrotic cells, and living cells respectively.

### Co‐Immunoprecipitation (CoIP)

Chondrocytes were lysed with cell lysis buffer (Beyotime, P0013) containing 1% PMSF. Forty micrograms of the protein lysate was used for the positive control group (input), and 1 mg of the protein lysate was used for the negative control group (IgG) and IP group. Twenty microliters of Protein A/G PLUS‐Agarose (Santa Cruz Biotechnology. sc‐2003) was added to the IgG group and IP group respectively and incubated for 1 h at 4 °C. Subsequently, protein lysate and agarose beads were separated by centrifugation at 1000 g for 5 min to remove nonspecifically bounded proteins. Twenty microliters agarose beads were added to the pretreated protein lysate and incubated with 4 µg Rabbit control IgG antibody (Abclonal, AC005), Anti‐Lactylation antibody (PTMBIO, PTM‐1401RM), and UGDH antibody (Sino Biological, 200912‐T44) at 4 °C for 12 h in a rotator. The beads were then washed three times with cell lysis buffer. Finally, cell lysis buffer and sodium dodecyl sulfate loading buffer (Beyotime, P0015) were added and heated at 100 °C for 10 min to elute the target proteins from agarose beads. The obtained proteins could be used for further Western blot.

### Mass Spectrum (MS)

Chondrocytes were lysed with cell lysis buffer (Beyotime, P0013). CoIP experiments with Anti‐L‐Lactyl Lysine antibody (PTMBIO, PTM‐1401RM) were performed to extract lactylated proteins from chondrocytes lysate. The extracted proteins were subjected to SDS‐PAGE. Proteins strips were excised and incubated with 4 µL of 0.05 M TCEP (Sigma, 51805‐45‐9) solution at 60 °C for 1 h. Then 2 µL of 55 mm MMTS (Sigma, 2949‐92‐0) solution was added and kept in the dark at room temperature for 45 min. The protein solution was added to 10 KDa ultrafiltration tubes (PALL, MAP010C38) and centrifuged at 12 000 g for 20 min. Hundred microliters of 8 M Urea (Amresco, 0378) solution was added and centrifuged at 12 000 g for 20 min twice. Hundred microliters of 0.25 M TEAB (Sigma, 71‐91‐0) was added and centrifuged at 12 000 g for 20 min and repeated three times. Fifty microliters of 0.5 m TEAB and 2% trypsin Trypsin(Promega, V5111) were added and incubated overnight at 37 °C. The next day, 1% trypsin was added and incubated at 37 °C for 4 h. A new collection tube was replaced and the filtrate was collected by centrifugation. The extracted peptides were detected on Mass spectrometer Q Exactive (Thermo Scientific). Data were analyzed by Fitgene Biotech Co, Guangzhou, Guangdong, China.

### Alcian Blue Staining

As previously reported,^[^ ^46^ ^]^ chondrocytes were seeded in six‐wells plates and treated with relative stimulus. After stimulation, chondrocytes were washed with PBS three times and fixed with 4% paraformaldehyde at romm temperature for 15 min. Then, the cells were washed with PBS three times and stained with alcian blue (Sigma‐ Aldrich, B8438) at 37 °C for 30 min. Finally, the cells were washed with PBS three times for further analysis.

Mouse cartilage tissues were fixed in 4% paraformaldehyde (Biosharp, BL539A), decalcified in 0.5 µm EDTA, embedded in paraffin, and sectioned into 4 um slices. All slides were deparaffinized in xylene, rehydrated with graded ethanol, and stained with alcian blue (Sigma‐ Aldrich, B8438) at 37 °C for 30 min. After washing three times with PBS, the slides were stained with hematoxylin and sealed withe resin.

### Glycosaminoglycan (GAGs) Detection

The Glycosaminoglycan content of chondrocytes was measured as manufacture's instruction by using glycosaminoglycan ELISA Kit (FineTest, EH4235). Briefly, the cells were lysed with RIPA buffer (Beyotime, P0013B) containing 1% PMSF (Beyotime, ST505). Fifty microliters of standard or tested samples were Added to the corresponding wells, followed by the addition of 50 µL of biotin‐labeled GAG antibody working solution to each well. Incubated the plate at 37 °C for 45 min. Aspirated the liquid from the microplate wells and added 350 µL of wash buffer to each well. Repeated this washing step three times. Subsequently, 100 µL of HRP‐streptavidin working solution were added to each well. Incubated the plate again at 37 °C for 30 min. Washed the plate five times with wash buffer. Then, dispensed 90 µL of TMB substrate into each well and incubated the plate at 37 °C in the dark for 20 min. After that, added 50 µL of reaction stop solution to each well. Immediately read the OD values at 450 nm using a microplate reader.

### UGDH Activity Assay

As reported,^[^ ^47^ ^]^ UGDH catalyzed the conversion of UDP‐glucose (UDP‐Glc) to UDP‐glucuronic acid (UDP‐GlcA). This process accompanied by the conversion of NAD^+^ to NADH. The enzymatic activity of UGDH could be reflected by the conversion of NAD^+^ to NADH. After relative treatment, UGDH was extracted from chondrocytes by CoIP. The assays were carried out utilizing a 0.1 m Tris‐HCl buffer solution (maintained at pH 7.4), incorporating 40 µg UGDH from CoIP, 1 mm UDP‐Glc (QiYue BIO,117756‐22‐6), and 10 mm NAD^+^ (MedChemExpress, HY‐B0445) as essential components, for a duration of 1 min at room temperature. After the incubation, the production of NADH was measured as manufacture's instruction by using NAD^+^/NADH Assay Kit (Beyotime, S0175). The samples were heated at 60 °C for 30 min to remove the remaining NAD^+^. Ten microliters Chromogenic solution was incubated with 20 µL of standard or tested samples at 37 °C for 30 min. Then, the OD values at 450 nm were detected with a microplate reader. A standard concentration curve could be made by the OD values of standard samples. The NADH concentration of tested samples could be calculated with the standard concentration curve. The protein samples of CoIP were subjected to Western blot. The NADH concentration of tested samples should be normalized to the grayscale value of UGDH.

### UDP‐Glc and UDP‐GlcA Measurement

The concentrations of UDP‐Glc and UDP‐GlcA were measured with UDP‐Glc ELISA Kit (FANKEW, FK0207‐OB) and UDP‐GlcA ELISA Kit (FANKEW, FK0212‐OB). As manufacture's instruction, 1 × 10^6^ chondrocytes were collected with 200 µL PBS and crushed with ultrasonic waves at a frequency of 20 kHz and a power of 150 W for 6s. Fifty microliters standard or tested samples were added to the corresponding wells and incubated with 50 µL HRP‐UDP‐Glc or HRP‐UDP‐GlcA at 37 °C for 60 min.

Aspirated the liquid from the wells and added 200 µL of wash buffer to each well. Repeated this washing step three times. Then, dispensed 50 µL of chromogenic solution A and 50 µL of chromogenic solution B into each well and incubated the plate at 37 °C in the dark for 15 min. After that, 50 µL of reaction stop solution was added to each well. Immediately read the OD values at 450 nm with a microplate reader. A standard concentration curve could be made by the OD values of standard samples. The UDP‐Glc and UDP‐GlcA concentration of tested samples could be calculated with the standard concentration curve.

### Lactylation Sites Analysis of UGDH

Chondrocytes were lysed with cell lysis buffer (Beyotime, P0013). CoIP experiments with UGDH antibody (Sino Biological, 200912‐T44) were performed to extract UGDH protein from chondrocytes lysate. The extracted UGDH protein was subjected to SDS‐PAGE. UGDH strips were excised and lysed by trypsin. Lactylation sites of UGDH were detected by liquid chromatography (Thermo EASY‐nLC 1200) and mass spectrometry analysis (Thermo Scientific Q Exactive HFX). Data were analyzed by Protein Gene Biotech, Wuhan, Hubei, China.

### Quantitative Real‐Time Polymerase Chain Reaction (qRT‐PCR)

After relative treatments, chondrocytes were processed to isolate total cellular RNA utilizing the Trizol reagent (Takara Bio, 9108). Following this, the isolated RNA transcripts underwent conversion into complementary DNA (cDNA) with the PrimeScript RT reagent kit (Takara Bio, RR037A). Adhering strictly to the manufacturer's protocols, qRT‐PCR was then conducted using SYBR Premix Ex Taq, (Takara Bio, RR820A). The amplification process and fluorescence detection were executed on the 7500 Real‐Time PCR System manufactured by Applied Biosystems. To normalize the data, Actin served as the reference gene. The primer sequences specific to each gene under investigation are listed in Table S2 (Supporting Information).

### Chromatin Immunoprecipitation Assay (ChIP) and ChIP Sequencing (ChIP‐seq)

ChIP kit (Sigma‐Aldrich, 17–295) was used for ChIP assay as manufacturer's instructions. Briefly, chondrocytes were initially fixed with formaldehyde to promote crosslinking of proteins to chromatins. Subsequently, the cells were lysed, and the chromatins were fragmented into 100–200 bp through sonication. The fragmented chromatins were then pre‐cleared to remove non‐specific binding, followed by overnight incubation with a STAT1‐specific antibody (Proteintech, 10144‐2‐AP) to allow for the formation of immune complexes. IgG was used for negative control. These complexes were immunoprecipitated using Protein A agarose beads, and the beads were thoroughly washed to remove unbound material. Next, the cross‐links were reversed, proteins were digested, and the purified DNA was isolated. Finally, Analyzing DNA by qPCR to assess STAT1 binding at specific genomic loci. The primer sequences utilized for ChIP‐qPCR analysis are listed in Table S3 (Supporting Information). For ChIP‐seq, purified DNA was detected and analyzed by Ribo Bio, Guangzhou, China with high throughput sequencing.

### Dual Luciferase Reporter Assay

To investigate the interaction between STAT1 and the MAP3K8 gene promoter, a dual luciferase reporter assay was performed. First, plasmids containing the MAP3K8 promoter upstream and Firefly Luciferase (F‐Luc) reporter gene were constructed and purchased from IGE Biotech, Guangzhou, China. The plasmids were then co‐transfected with a Renilla Luciferase (R‐Luc) vector as an internal control into HEK293T cells with Lipofectamine 3000 (Invitrogen, L3000001). STAT1 vetor (control) and STAT1 plasmids (STAT1 OE), constructed by IGE Biotech, Guangzhou, China, were also co‐transfected to evaluate the effect of STAT1 on the MAP3K8 promoter. HEK293T cells were then incubated under standard conditions to allow protein‐DNA interactions. Following transfection and incubation, HEK293T cells were lysed, and F‐Luc and R‐Luc activities were sequentially measured using appropriate substrates in a luminometer. The relative light units of F‐Luc were normalized to those of R‐Luc to account for differences in transfection efficiency and expression levels. Finally, the normalized F‐Luc activity in cells co‐transfected with STAT1 OE was compared to STAT1 vetor to assess the impact of STAT1 binding on MAP3K8 promoter activity.

### SiRNA and Plasmid Transfection

All siRNA and plasmid were constructed and purchased from IGE Biotech, Guangzhou, China as listed in Table S4 (Supporting Information). None of the siRNAs used in this study were fluorescently labeled. For siRNA transfection, chondrocytes were transfected with UGDH siRNA (si‐UGDH) and negative control (NC) by using Lipofectamine 3000 (Invitrogen, L3000001). After transfection, chondrocytes were treated with or without IL‐1β (10 ng ml^−1^) and DCA (20 mm) for 24 h. For plasmid transfection, Overexpression of flag‐tagged wild type UGDH (UGDH WT), 6^th^ lysine mutated into arginine of UGDH (UGDH K6R) and 399^th^ lysine mutated into arginine of UGDH (UGDH K399R) were introduced into chondrocytes by using Lipofectamine 3000 (Invitrogen, L3000001) with or without the pretreatments of IL‐1β and UGDH siRNA. After 24 h, chondrocytes were collected for further experiments.

### Separation of Nuclear and Cytoplasmic Proteins

Separation of nuclear and cytoplasmic proteins was conducted by using Nuclear and Cytoplasmic Protein Extraction Kit (Beyotime, P0027). As manufacture's instruction, chondrocytes were washed with PBS and collected by cell scrapers. A ten‐fold volume of cytosolic protein extraction reagent A containing 1% PMSF was added at 4 °C for 15 min. After that, 10 µL of cytosolic protein extraction reagent B were added and ice bath for 1 min. The mixture was shaken violently for 5 s and centrifuged at 12000 g at 4 °C for 5 min. The supernatant was the extracted cytoplasmic protein lysate. Fifty microliters of nuclear protein extraction reagent containing 1% PMSF were added to resuspend the precipitation and incubated at 4 °C for 30 min. After incubation, the mixture was centrifuged at 12000 g at 4 °C for 10 min to extract the nuclear proteins.

### Quantification and Statistical Analysis

Results were reported as the mean ± standard deviation (SD) or mean ± 95% confidence interval (CI). All data were derived from at least three independent experiments. Comparisons between two groups were analyzed by Student's t‐test. One‐way analysis of variance (ANOVA) with Dunnett's post hoc test was conducted for comparisons among multiple groups. P value <0.05 was represented statistically significant. GraphPad Prism 7.0 was used for all the data statistical analyses.

## Conflict of Interest

The authors declare no conflict of interest.

## Author Contributions

W.L. and X.C. contributed equally to this work. C.D. and S.T. designed and funded this project. W.L., H.Y., and X.N. were responsible for cell experiments. J.R., J.K., X.N., and Y.C. were responsible for animal experiments. X.C. and W.L. were responsible for writing and revising this article. W.L. was responsible for data analysis. All authors have reviewed the article.

## Supporting information



Supporting Information

## Data Availability

The data that support the findings of this study are available in the supplementary material of this article.
