# Supplementary material for: UGDH Lactylation Aggravates Osteoarthritis by Suppressing Glycosaminoglycan Synthesis and Orchestrating Nucleocytoplasmic Transport to Activate MAPK Signaling
Source: Adv Sci (Weinh). 2025 Mar 27;12(20):2413709. doi: 10.1002/advs.202413709 (PMC12120796; doi:10.1002/advs.202413709)
Supplement: Supplementary file 1 — Supporting Information [file ADVS-12-2413709-s001.docx]

**Table S1. Information of non-OA and OA patients whose synovial fluid was used for lactate detection.**

| **NO.** | **Gender** | **Age** | **NO.** | **Gender** | **Age** |
| --- | --- | --- | --- | --- | --- |
| Non-OA1 | Female | 51 | OA1 | Female | 57 |
| Non-OA2 | Female | 67 | OA2 | Female | 59 |
| Non-OA3 | Female | 54 | OA3 | Male | 74 |
| Non-OA4 | Male | 49 | OA4 | Female | 49 |
| Non-OA5 | Female | 52 | OA5 | Male | 63 |
| Non-OA6 | Male | 57 | OA6 | Male | 58 |
| Non-OA7 | Male | 62 | OA7 | Female | 56 |
| Non-OA8 | Male | 44 | OA8 | Female | 62 |
| Non-OA9 | Female | 57 | OA9 | Male | 48 |
| Non-OA10 | Female | 56 | OA10 | Female | 70 |

**Table S2. Primers used in qRT-PCR experiments.**

| **Gene** | **Forward primer (5’-3’)** | **Reverse primer (5’-3’)** |
| --- | --- | --- |
| GAPDH  BDNF  IL1A  MET  MAP3K8 | GGAGCGAGATCCCTCCAAAAT  TAACGGCGGCAGACAAAAAGA  TGGTAGTAGCAACCAACGGGA  GGTTCACTGCATATTCTCCCC  ATGGAGTACATGAGCACTGGA | GGCTGTTGTCATACTTCTCATGG  TGCACTTGGTCTCGTAGAAGTAT  ACTTTGATTGAGGGCGTCATTC  ACCATCTTTCGTTTCCTTTAGCC  GCTGGCTCTTCACTTGCATAAAG |

**Table S3. Primers used in ChIP-qPCR experiments.**

| **Gene** | **Forward primer (5’-3’)** | **Reverse primer (5’-3’)** |
| --- | --- | --- |
| IL1A | GTTGTTCACAGTCCCAGAAAAGCG | AACTAGGGTGGGTGAGGTTCAGAG |
| MAP3K8 | CCACAGTCGCGGTTCCAAGAAG | GGGAGGGCAGTCACCAGACC |

**Table S4. Sequence of siRNA.**

| **Oligo** | **Sense (5’-3’)** | **Antisense (5’-3’)** |
| --- | --- | --- |
| NC | UUCUCCGAACGUGUCACGUdTdT | ACGUGACACGUUCGGAGAAdTdT |
| UGDH | CCCGGAUCAUAGAUAGUCUdTdT | AGACUAUCUAUGAUCCGGGdTdT |
| STAT1 | CUGACUUCCAUGCGGUUGAdTdT | UCAACCGCAUGGAAGUCAGdTdT |


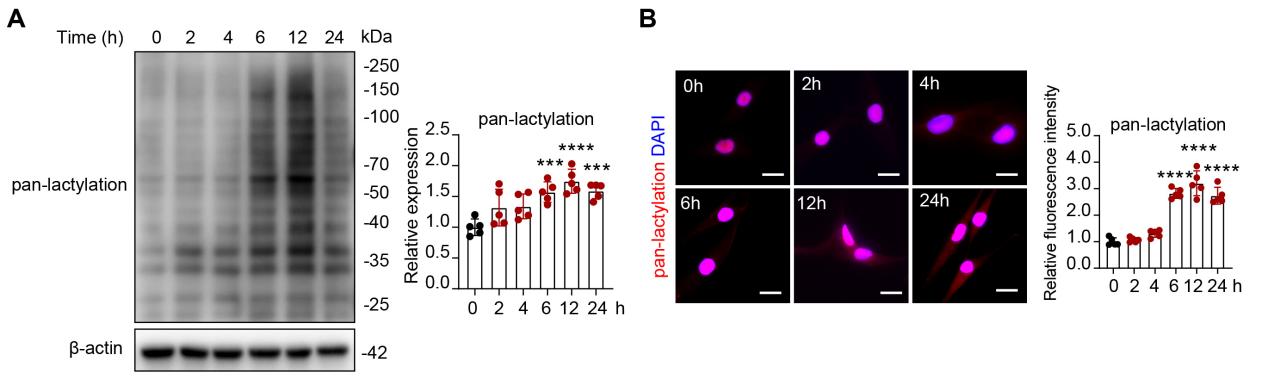


**Figure S1.**

Expression of pan-lactylation in chondrocytes with IL-1β treatment. A) Western blot analysis of pan-lactylation in chondrocytes with IL-1β (10 ng/ml) treatment for 0, 2, 4, 6, 12 and 24 h (n=5). The data were normalized to β-actin. B) Immunofluorescence staining of pan-lactylation in chondrocytes with IL-1β (10 ng/ml) treatment for 0, 2, 4, 6, 12 and 24 h (n=5), Scale bar, 25 μm. Data were presented as mean ±SD and analyzed by One-way analysis of variance (ANOVA) with Dunnett’s post hoc test (A,B). N represents the number of independent repeated experiments. NS: no significance, *: *P*<0.05, **:*P*<0.01, ***:*P*<0.001, ****:*P*<0.0001.


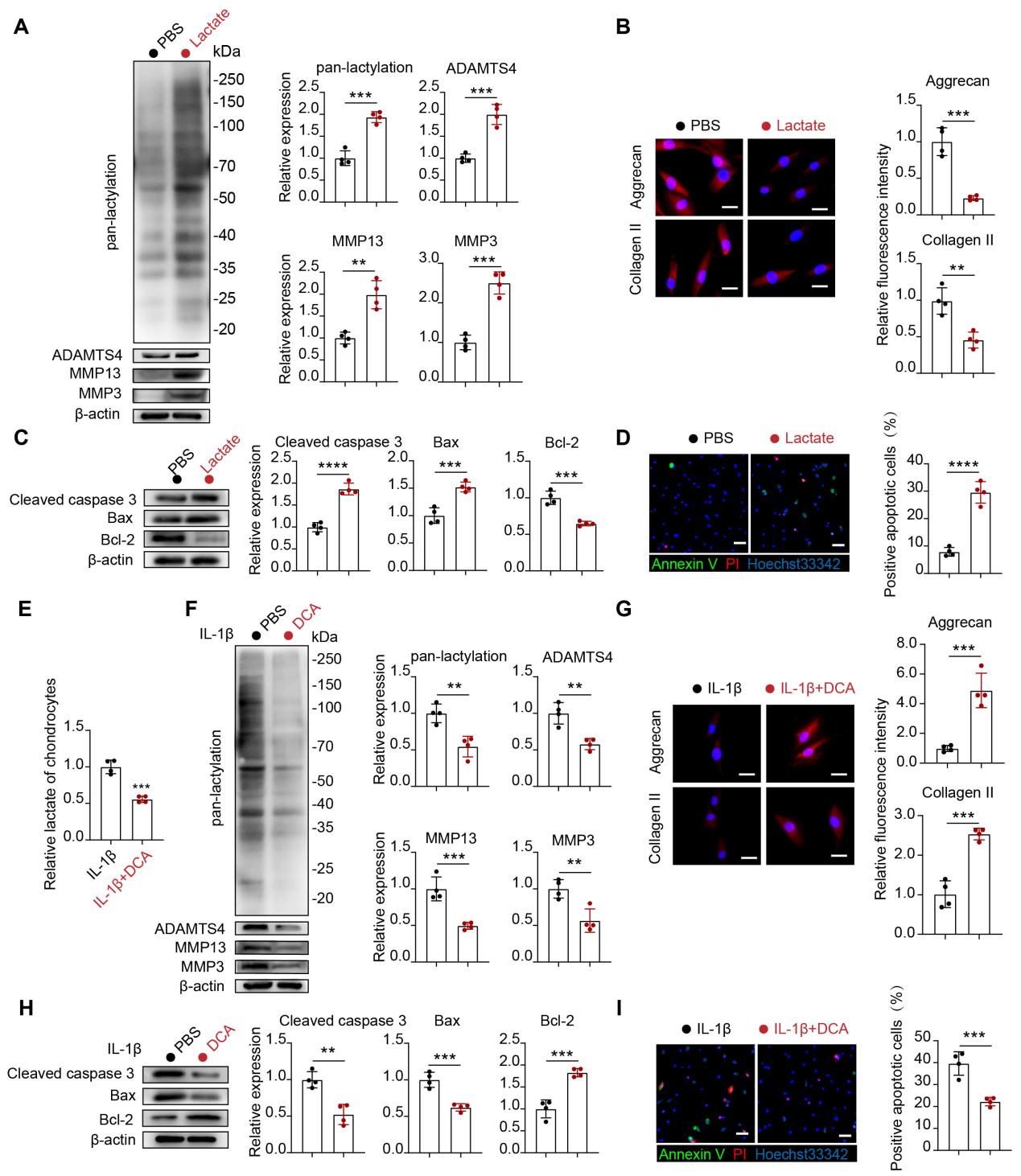


**Figure S2.**

Hyper-lactylation aggravates extracellular matrix (ECM) degradation and chondrocyte apoptosis. A) Western blot analysis of pan-lactylation, MMP3, MMP13 and ADAMTS4 in chondrocytes with lactate (25 mM) treatment for 24 h (n=4). The data were normalized to β-actin. B) Immunofluorescence staining of Collagen II and Aggrecan in chondrocytes with lactate (25 mM) treatment for 24 h (n=4). Scale bar, 25 μm. C) Western blot of cleaved-caspase 3, Bax and Bcl-2 in chondrocytes with lactate (25 mM) treatment for 24 h (n=4). The data were normalized to β-actin. D) Representative immunofluorescence images of Annexin V (green) and PI (red) in chondrocytes with lactate (25 mM) treatment for 24 h (n=4), Scale bar, 100 μm. E) Relative lactate of chondrocytes with DCA (20 mM) treatment in the presence of IL-1β. F) Western blot of pan-lactylation, MMP3, MMP13 and ADAMTS4 in chondrocytes with DCA (20 mM) treatment in the presence of IL-1β (n=4). The data were normalized to β-actin. G) Immunofluorescence staining of Collagen II and Aggrecan in chondrocytes with DCA (20 mM) treatment in the presence of IL-1β (n=4). Scale bar, 25 μm. H) Western blot of cleaved-caspase 3, Bax and Bcl-2 in chondrocytes with DCA (20 mM) treatment in the presence of IL-1β (n=4). The data were normalized to β-actin. I) Representative immunofluorescence images of Annexin V (green) and PI (red) in chondrocytes with DCA (20 mM) treatment in the presence of IL-1β (n=4), Scale bar, 100 μm. Data were presented as mean ±SD and analyzed by Student’s t-test (A-I). N represents the number of independent repeated experiments. NS: no significance, *:*P*<0.05, **:*P*<0.01, ***:*P*<0.001, ****:*P*<0.0001.


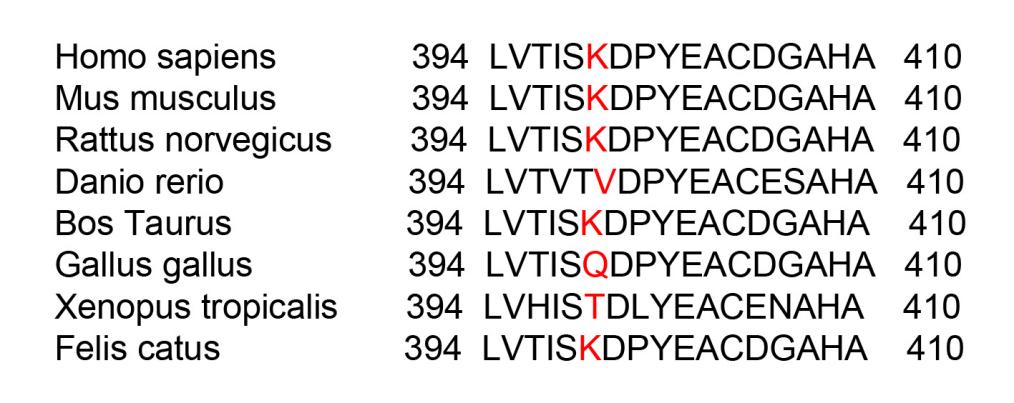


**Figure S3.**

K399 site (marked as red) of UGDH is not highly conserved. The sequences (394^th^-410^th^ amino acids) around UGDH K399 site from different species were aligned.


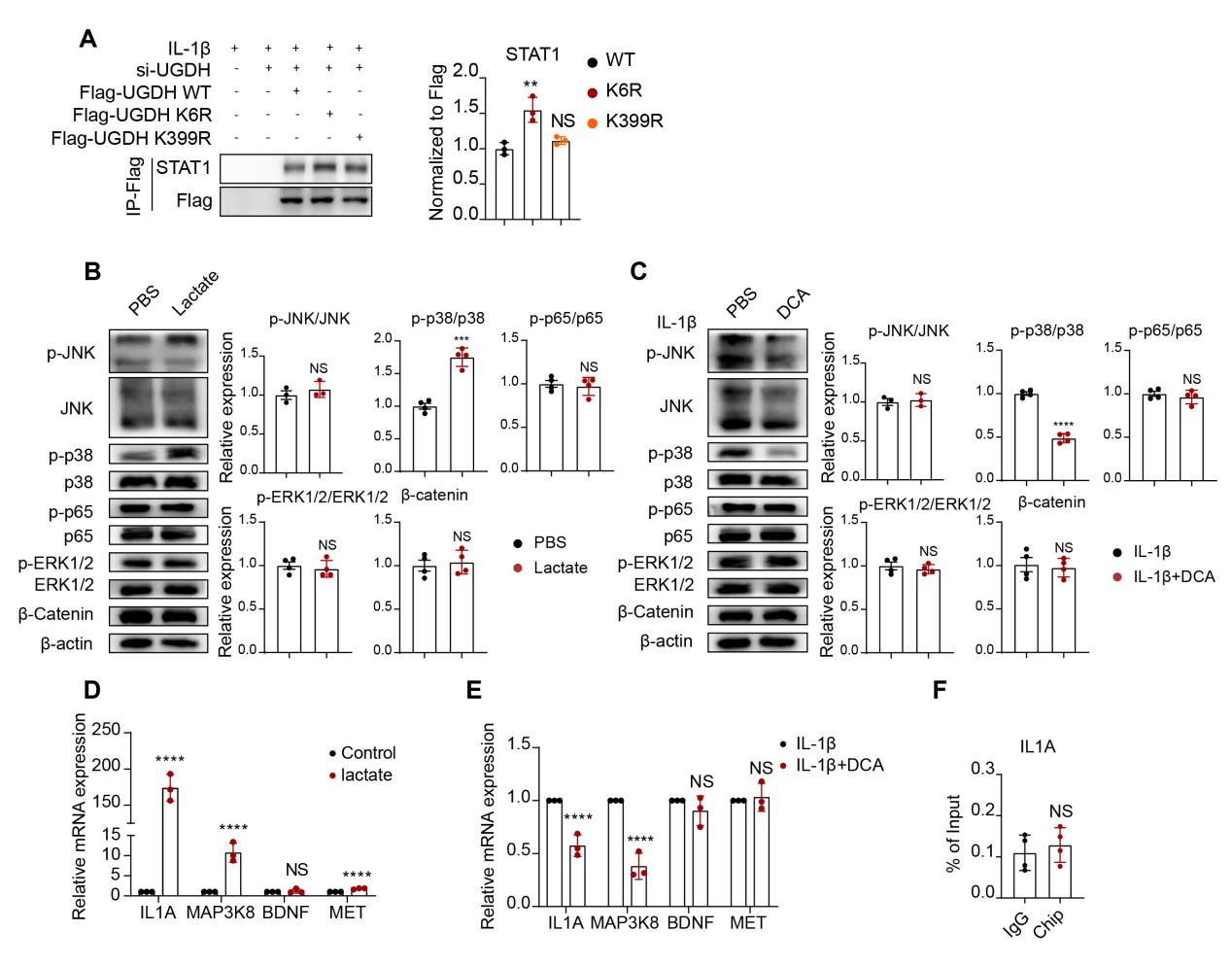


**Figure S4.**

A) Interaction assay of Flag-UGDH with STAT1 in IL-1β treated chondrocytes (Flag-UGDH WT, Flag-UGDH K6R, Flag-UGDH K399R) (n=4). COIP was conducted with Anti-Flag antibody. The data were normalized to Flag. B) Western blot analysis of marker phosphorylated proteins of NF-κB, MAPK and WNT pathway in chondrocytes with lactate (25 mM) treatment for 24 h (n=4). C) Western blot analysis of phosphorylated proteins of NF-κB, MAPK and WNT pathway in chondrocytes with DCA (20 mM) treatment in the presence of IL-1β (n=4). D) Relative mRNA expression of IL1A, MAP3K8, BDNF and MET in chondrocytes under the stimulation of lactate (25 mM) (n=3). E) Relative mRNA expression of IL1A, MAP3K8, BDNF and MET in chondrocytes with DCA (20 mM) treatment in the presence of IL-1β (n=3). F) Relative quantification of ChIP-qPCR for IL1A. N represents the number of independent repeated experiments. All data were presented as mean ±SD and analyzed by Student’s t-test. NS: no significance, *: *P*<0.05, **:*P*<0.01, ***:*P*<0.001, ****:*P*<0.0001.


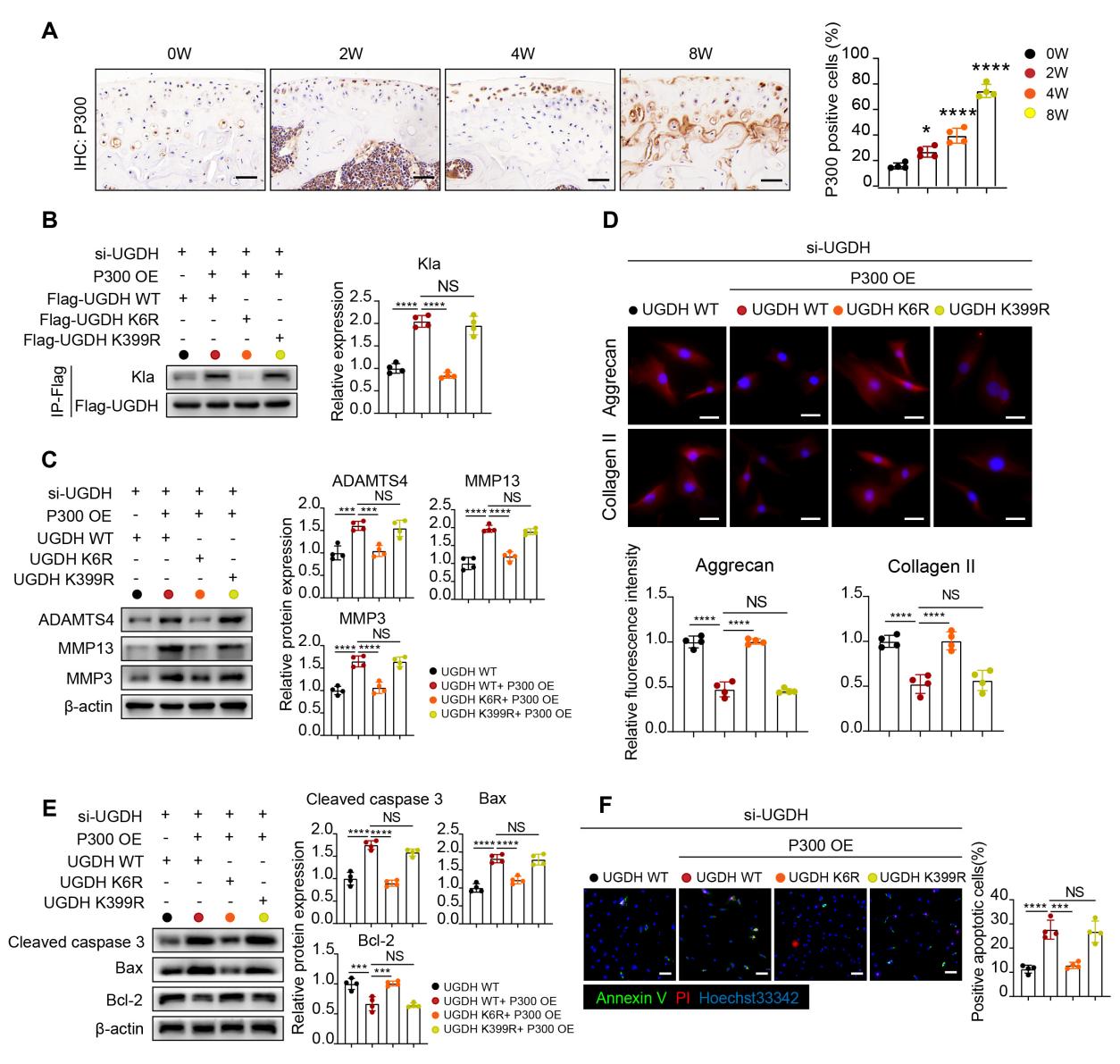


**Figure S5.**

Effects of UGDH mutation on ECM degradation and chondrocyte apoptosis to P300 overexpression. A) Immunohistochemistry (IHC) of P300 in the articular cartilage from DMM-induced OA mice at 0,2,4,8 W (n=4 per group). Scale bars, 50 μm. B) Immunoprecipitation and western blot analysis of Kla expression in chondrocytes with Flag-UGDH wild type (WT), K6R, and K399R mutants, with or without P300 overexpression (OE). Relative Kla expression normalized to Flag-UGDH (n=4). C) Western blot analysis of MMP3, MMP13 and ADAMTS4 in the same conditions as in B, with quantification normalized to β-actin (n=4). D) Immunofluorescence staining of Aggrecan and Collagen II in chondrocytes (n=4). Scale bar, 25 μm. E) Western blot analysis of cleaved caspase 3, Bax, and Bcl-2 expression with quantification normalized to β-actin (n=4). F) Representative immunofluorescence images of Annexin V (green) and PI (red) in chondrocytes, with quantification of apoptotic cell percentage (n=4). Scale bar, 100 μm. All data were presented as mean ± SD and analyzed by One-way analysis of variance (ANOVA) with Dunnett’s post hoc test. N represents the number of independent repeated experiments. NS: no significance, *:*P*<0.05, **:*P*<0.01, ***:*P*<0.001, ****:*P*<0.0001.


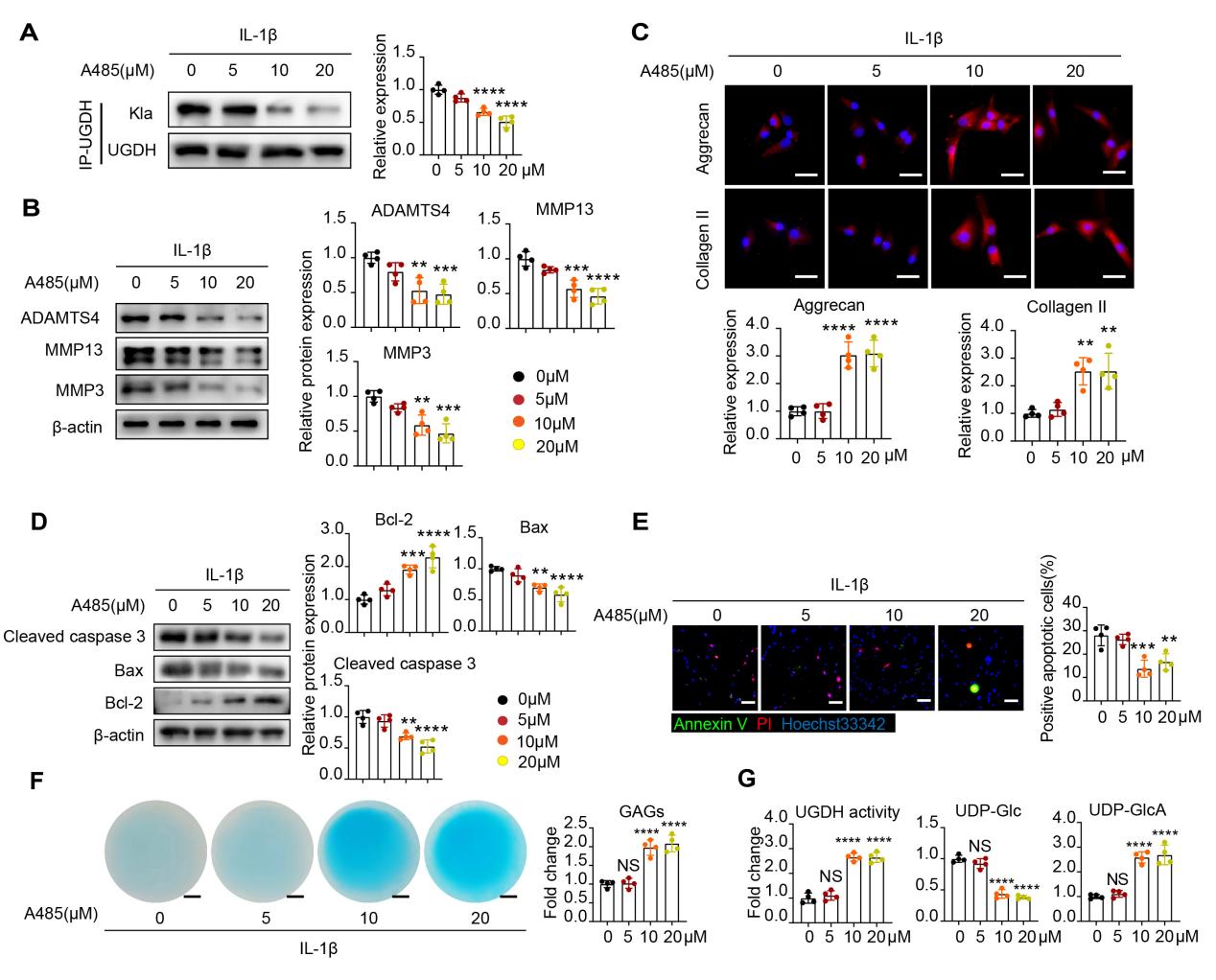


**Figure S6.**

Effects of A485 on ECM degradation and chondrocyte apoptosis. A) Western blot of lactylated UGDH in chondrocytes with A485 (0,5,10,20 μM) treatment in the presence of IL-1β (10 ng/ml). The data were normalized to UGDH. B) Western blot of MMP3, MMP13 and ADAMTS4 in chondrocytes with A485 (0,5,10,20 μM) treatment in the presence of IL-1β (10 ng/ml). The data were normalized to β-actin. C) Immunofluorescence staining of Collagen II and Aggrecan in chondrocytes with A485 (0,5,10,20 μM) treatment in the presence of IL-1β (10 ng/ml). Scale bar, 25 μm. D) Western blot of cleaved-caspase 3, Bax and Bcl-2 in chondrocytes with A485 (0,5,10,20 μM) treatment in the presence of IL-1β (10 ng/ml). The data were normalized to β-actin. E) Representative immunofluorescence images of Annexin V (green) and PI (red) in chondrocytes with A485 (0,5,10,20 μM) treatment in the presence of IL-1β (10 ng/ml), Scale bar, 100 μm. F) Alcian blue staining and ELISA assay of GAGs in chondrocytes with A485 (0,5,10,20 μM) treatment in the presence of IL-1β (10 ng/ml). G) UGDH activity, UDP-Glc and UDP-GlcA concentrations measurements in chondrocytes with A485 (0,5,10,20 μM) treatment in the presence of IL-1β (10 ng/ml). All data were presented as mean ± SD and analyzed by One-way analysis of variance (ANOVA) with Dunnett’s post hoc test. N represents the number of independent repeated experiments. NS: no significance, *:*P*<0.05, **:*P*<0.01, ***:*P*<0.001, ****:*P*<0.0001.


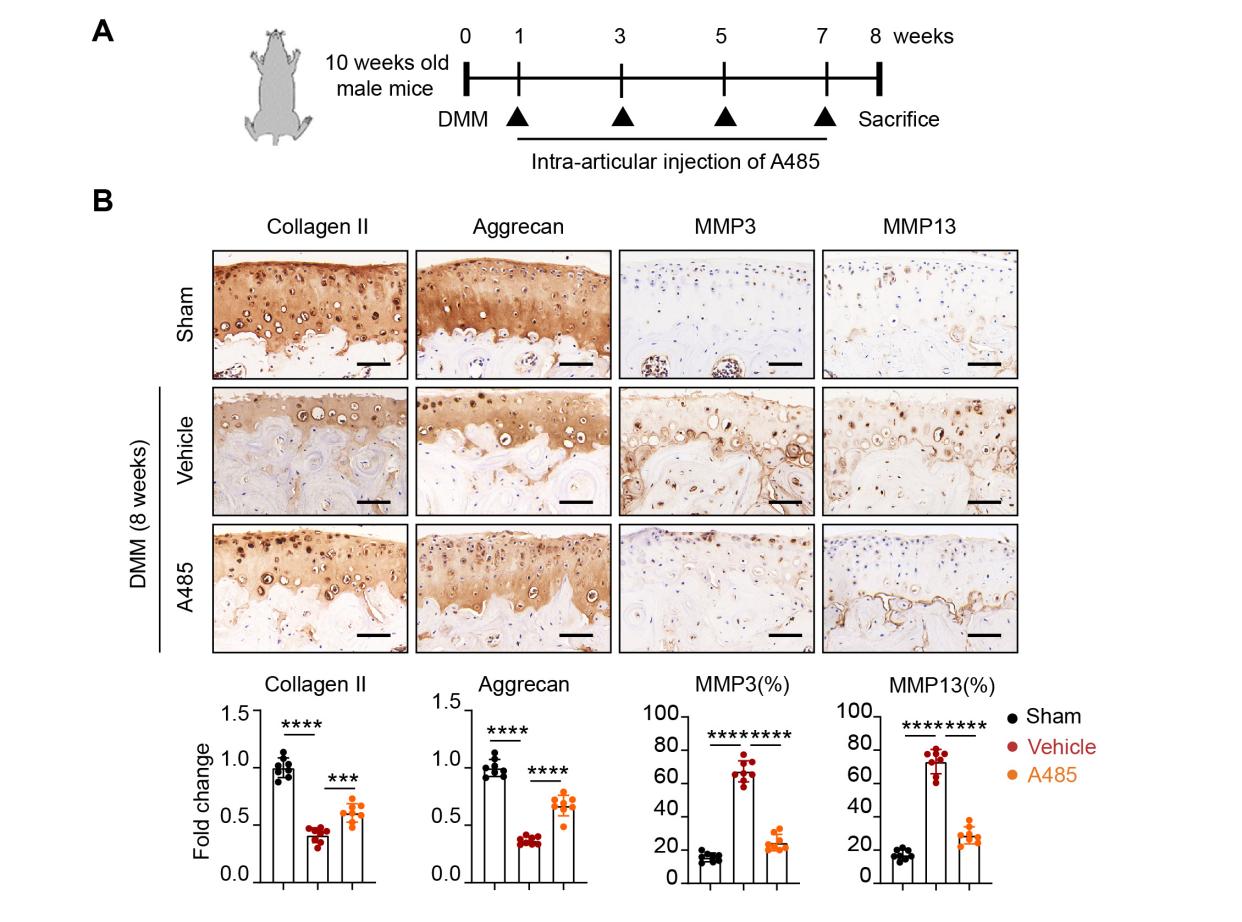


**Figure S7.**

A) Schematic diagram of animals experiment design. B) IHC of Collagen II, Aggrecan, MMP3 and MMP13 of articular cartilage in sham, DMM induced OA mice with A485 or DMSO. (n=8 per group). Scale bars, 50 μm. All data were presented as mean ± SD and analyzed by One-way analysis of variance (ANOVA) with Dunnett’s post hoc test. N represents the number of sample size. NS: no significance, *:*P*<0.05, **:*P*<0.01, ***:*P*<0.001, ****:*P*<0.0001.
